# Real-world pharmacovigilance assessment of meropenem-associated adverse events: age stratification and time-to-onset analysis using FAERS

**DOI:** 10.3389/fmed.2026.1890727

**Published:** 2026-08-12

**Authors:** Xiaobin Luo, Yali Zhong, Yong Zou, Chenghao Yang

**Affiliations:** 1 Department of Neurosurgery, Zigong Fourth People's Hospital, Zigong, Sichuan, China; 2 Department of Neurosurgery, The Affiliated Hospital of Southwest Medical University, Luzhou, China; 3 Department of Gastroenterology, Zigong First People's Hospital, Zigong, Sichuan, China

**Keywords:** meropenem, FAERS, age stratification, real-world evidence, time-to-onset analysis

## Abstract

**Background:**

Meropenem is a broad-spectrum carbapenem antibiotic widely used for severe infections; however, its real-world adverse event profile and age-stratified pharmacovigilance signals remain insufficiently characterized. This study aimed to evaluate signals of meropenem-associated adverse events using the FDA Adverse Event Reporting System (FAERS).

**Methods:**

FAERS reports from 2004 Q1 to 2025 Q4 were extracted. Reports listing meropenem as the primary suspect drug were included after standardization and deduplication. Disproportionality analyses were performed at the Preferred Term (PT) level using the reporting odds ratio, proportional reporting ratio, empirical Bayes geometric mean, and information component, with a Bonferroni correction applied to PRR chi-square *P*-values for multiple PT-level comparisons. Reports were stratified into five age groups: < 18, 18–39, 40–59, 60–79, and ≥80 years. Age-, sex-, seriousness-, fatality-, and time-to-onset stratified analyses were conducted. A healthcare professional (HCP)-only subset analysis was performed using the same statistical framework.

**Results:**

A total of 5,018 meropenem primary suspect drug-event records were identified, corresponding to 4,431 deduplicated report-level cases after merging FAERS DEMO, DRUG, and REAC tables. Overall, 254 PT-level signals remained significant after four-algorithm screening and Bonferroni correction. These signals were mainly distributed across infections and infestations, investigations, skin and subcutaneous tissue disorders, blood and lymphatic system disorders, and hepatobiliary disorders. Frequently detected PTs included pyrexia, acute kidney injury, pancytopenia, thrombocytopenia, septic shock, seizure, DRESS, multiple organ dysfunction syndrome, and sepsis. Age-stratified analysis showed a heterogeneous distribution of signals across age groups, with higher numbers of reports and signals observed in the 40–79-year age group. The proportion of reported serious outcomes increased across age strata, exceeding 85% among patients aged ≥40 years. Median time-to-onset was 5 days, and the Weibull shape parameter (β = 0.72) suggested an early-reporting pattern of events. HCP-only analysis demonstrated consistent signal patterns compared with the primary analysis at the PT level.

**Conclusions:**

Meropenem-associated adverse event signals involved multiple organ systems and demonstrated heterogeneous distribution across age groups. Early-onset clustering was observed for several key signals. These findings represent FAERS-based disproportionality signals rather than causal associations and require further sclinical validation.

## 1 Introduction

Meropenem is a broad-spectrum carbapenem antibiotic with potent antimicrobial activity against Gram-negative and Gram-positive bacteria, as well as anaerobic organisms. It plays an important role in the treatment of multidrug-resistant Gram-negative infections. It is widely used for complicated skin and soft tissue infections (cSSTIs), complicated intra-abdominal infections, hospital-acquired pneumonia, and other serious infections caused by resistant pathogens (1). In clinical practice, carbapenems are considered key therapeutic options for severe infections requiring broad-spectrum coverage, and meropenem has demonstrated favorable efficacy and tolerability in cSSTIs and secondary peritonitis (1, 2). It is also frequently used in hospital-acquired infections and intensive care unit (ICU) settings, where it is among the most commonly prescribed broad-spectrum antibiotics in acute care hospitals (1).

In surgical and perioperative settings, meropenem is commonly used for early postoperative infections, complicated infections, and empirical therapy in critically ill patients. It has also been reported to have potential value in biofilm-associated surgical site infections (3). Due to its ability to cross the blood-brain barrier, meropenem reaches high concentrations in cerebrospinal fluid and is effective against bacterial meningitis (4). Clinical applications have also been reported in central nervous system infections caused by resistant organisms, including carbapenem-resistant Acinetobacter baumannii (5). In addition, meropenem is widely used for pediatric bacterial meningitis and multidrug-resistant Gram-negative infections due to its relatively low risk of seizures and favorable safety profile (6, 7).

Although meropenem is generally well-tolerated, its clinical use is complicated by multiple factors. In critically ill patients, altered renal function, volume of distribution, and inflammatory status may affect attainment of pharmacokinetic/pharmacodynamic (PK/PD) targets, potentially influencing drug exposure (8). In addition, broad-spectrum antibiotics may disrupt gut microbiota and vitamin K metabolism and have been associated with coagulation abnormalities in specific populations, such as patients with severe burns (9). Emerging evidence and case reports suggested that meropenem may be associated with neurotoxicity, renal dysfunction, hematologic abnormalities, hypersensitivity reactions, and other adverse events (10–15). Among these, neurotoxicity may manifest as seizures, encephalopathy, or disturbances of consciousness, whereas hematologic adverse events may present as thrombocytopenia, neutropenia, or pancytopenia. These adverse reactions may be particularly pronounced in critically ill patients, those with impaired renal function, and individuals receiving multiple concomitant medications.

Age is an important determinant of antimicrobial safety. As the population ages, the proportion of older adults in surgical and ICU settings continues to increase. Older patients are more likely to experience physiological decline, reduced renal function, comorbidities, and polypharmacy, which may increase vulnerability to drug-related adverse events (16). At the same time, older adults are more frequently exposed to meropenem due to higher infection severity and may therefore require closer safety monitoring during treatment (17). However, systematic evidence on age-related differences in the safety profiles of meropenem remains limited. Existing studies on meropenem safety have primarily been derived from FAERS-based signal detection, randomized controlled trials, and observational studies (18–20). Although these studies provided important insights, they are limited by the underrepresentation of elderly populations, the limited detection of rare adverse events, and the insufficient characterization of temporal patterns. In particular, previous studies have largely focused on overall adverse event profiles. In contrast, comparatively few have systematically evaluated the real-world distribution patterns of meropenem-associated adverse events across age strata, system-organ class classifications, and time-to-onset characteristics.

Although randomized controlled trials (RCTs) are the gold standard for evaluating drug efficacy and safety, they are limited in detecting rare or delayed adverse events due to strict eligibility criteria, limited sample sizes, and short follow-up durations. Furthermore, elderly and critically ill patients with multimorbidity and polypharmacy are often underrepresented in clinical trials, despite being major recipients of meropenem therapy. Consequently, real-world patterns of drug exposure and adverse event profiles in these populations may not be fully captured in clinical trials. Large-scale pharmacovigilance databases based on spontaneous reporting systems provide an important approach to addressing these evidence gaps. The United States Food and Drug Administration Adverse Event Reporting System (FAERS), characterized by broad population coverage and diverse reporting sources, facilitates the identification of potential safety signals that may not be fully captured in clinical trials and serves as an important complement to conventional clinical research. Within pharmacovigilance research, disproportionality analysis enables the exploration of statistical associations between specific drugs and adverse events using real-world reports, whereas time-to-onset (TTO) analysis and Weibull distribution models further characterize the temporal patterns of adverse event occurrence, thereby providing complementary evidence for identifying an early exposure monitoring window and optimizing clinical risk management strategies.

Accordingly, the present study systematically evaluated the real-world safety characteristics of meropenem-associated adverse events using the FAERS database, with particular emphasis on signal distribution across age groups, system organ class characteristics, serious outcomes, and temporal dynamics. By integrating age-stratified disproportionality analysis with TTO and Weibull-based time-dynamic modeling, this study aimed to provide a comprehensive real-world pharmacovigilance profile of meropenem.

## 2 Materials and methods

### 2.1 Data source and study design

This retrospective pharmacovigilance study was conducted using the United States Food and Drug Administration Adverse Event Reporting System (FAERS). Publicly available FAERS reports from the first quarter of 2004 to the fourth quarter of 2025 were included. The FAERS database primarily comprises the demographic information table (DEMO), drug information table (DRUG), adverse reaction table (REAC), therapy start and end date table (THER), report source table (RPSR), and outcome table (OUTC). Reports were used as the fundamental analytical unit, and data linkage across tables was performed using the unique report identifier (PRIMARYID).

**Figure 1:**
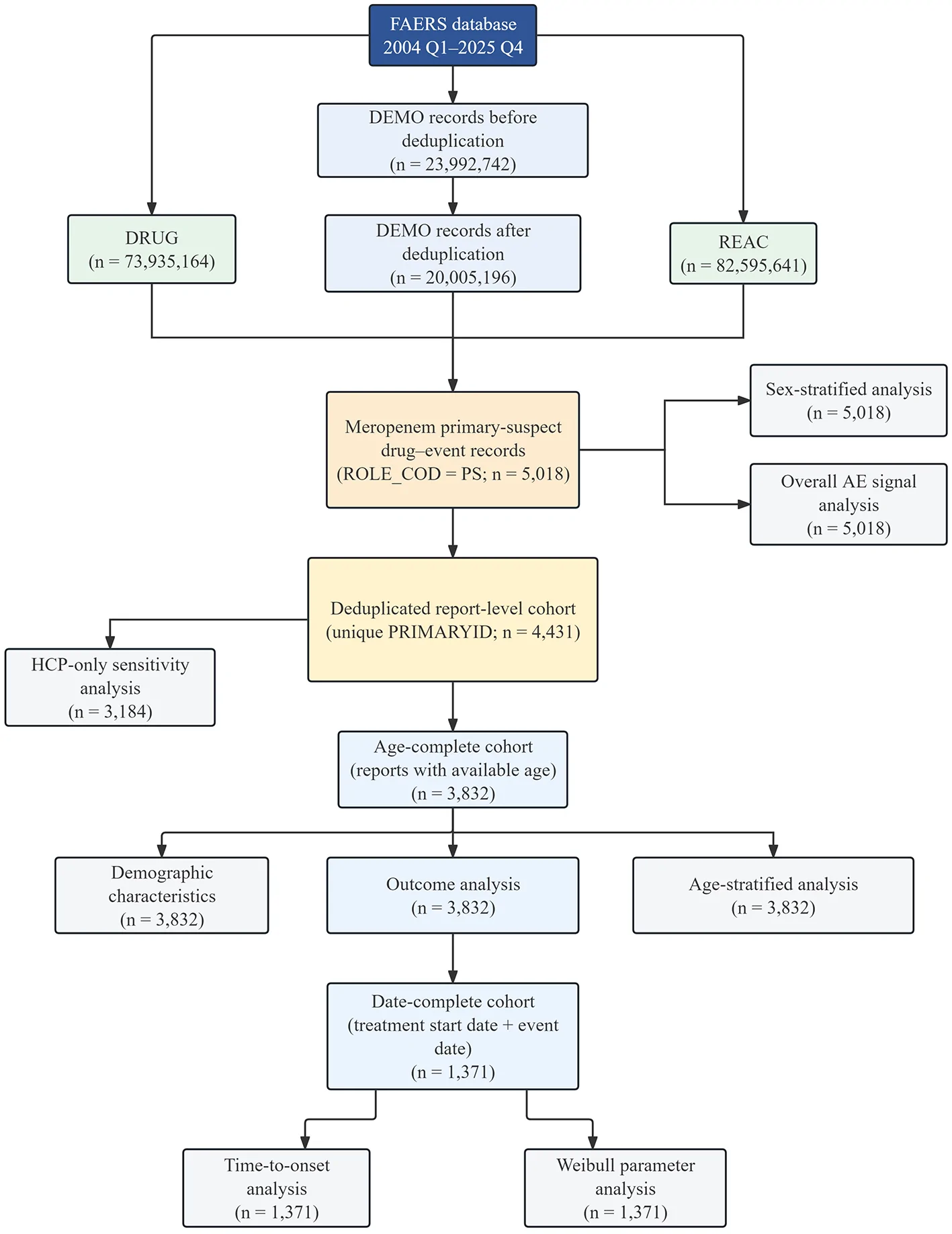
Study workflow for FAERS-based analysis of meropenem adverse event signals. The workflow shows data extraction, deduplication, identification of meropenem primary suspect reports, construction of the report-level analytical cohort, and subsequent analyses, including overall signal detection, sex-stratified analysis, age-stratified analysis, outcome analysis, healthcare professional-only sensitivity analysis, time-to-onset analysis, and Weibull parameter analysis. FAERS, FDA Adverse Event Reporting System.

A total of 5,018 meropenem-related drug–event records were extracted from the DRUG table where meropenem was identified as the primary suspect (ROLE_COD = PS). These records represent Preferred Term (PT)-level drug–event pairs, as a single FAERS report may contain multiple adverse events. Each PT was counted once per report to avoid duplication. This dataset was used for overall signal detection and sex-stratified analyses based on report-level demographic information linked via PRIMARYID. Subsequently, the DEMO, DRUG, and REAC tables were merged using PRIMARYID and deduplicated at the report level, resulting in 4,431 unique meropenem-associated reports for the primary analysis. Based on this primary cohort, three analytical subsets were derived: (1) Healthcare professional (HCP) sensitivity cohort: 3,184 reports submitted by healthcare professionals (e.g., physicians, pharmacists, and other clinical reporters) were included for sensitivity analysis to assess the robustness of the primary findings. (2) Age-stratified cohort: Among the 4,431 deduplicated reports, cases with complete age information were selected, yielding 3,832 reports for demographic characterization, outcome analysis, and age-stratified analyses. (3) Time-to-onset (TTO) cohort: A total of 1,371 reports containing complete treatment initiation and adverse event onset dates were included for TTO and Weibull parameter analyses. All analyses were conducted at predefined analytical levels, with PT-level analyses used for disproportionality signal detection and report-level analyses used for demographic, stratified, and time-to-onset assessments. The data screening workflow is presented in Figure 1.

### 2.2 Drug and adverse event definitions

The target drug was meropenem. Meropenem-associated reports were identified in the DRUG table through standardized drug name normalization, and only reports in which meropenem was designated as the primary suspect drug were included to improve the specificity of drug–event association analyses. Adverse events were extracted using preferred terms (PTs) recorded in the REAC table and further categorized into the system organ class (SOC) hierarchy. Both PT and SOC terminology followed the Medical Dictionary for Regulatory Activities (MedDRA) classification system. To minimize the impact of duplicate reporting, report-level analyses were conducted using unique PRIMARYID values as the unit of analysis. For PT-level analyses, repeated occurrences of identical PTs within the same report were counted only once.

### 2.3 Age stratification, outcome definitions, and sensitivity analysis

Based on reported ages, cases were classified into five age groups: 0–17.99, 18–39.99, 40–59.99, 60–79.99, and ≥80 years. A total of 3,832 reports contained valid age information and were included in age-stratified analyses. Outcome information was extracted from the OUTC table. Serious outcomes included death, hospitalization, life-threatening conditions, disability, and other medically serious events. Death was additionally analyzed separately as an indicator of fatal burden. To evaluate the influence of reporting source on the primary findings, an additional sensitivity analysis restricted to healthcare professional (HCP) reports was performed. This HCP-only cohort comprised 3,184 reports. Healthcare professional (HCP) reports were identified using the reporter occupation variable OCCP_COD in the FAERS DEMO table. Reports with OCCP_COD coded as MD (physician), PH (pharmacist), HP (health professional), or OT (other health professional) were classified as HCP reports. Reports coded as CN (consumer) or LW (lawyer), as well as reports with missing OCCP_COD, were excluded from the HCP-only sensitivity analysis. This subset was used to assess the robustness of the primary disproportionality findings by reducing potential reporting bias associated with non-professional reporting sources.

### 2.4 Disproportionality analysis and signal detection criteria

This study employed both frequentist and Bayesian disproportionality methods to detect potential drug–adverse event signals, including the reporting odds ratio (ROR), the proportional reporting ratio (PRR), the empirical Bayes geometric mean (EBGM), and the information component (IC). Disproportionality analyses were conducted at the drug–Preferred Term (PT) level using report-level data. For each drug–PT pair, a 2 × 2 contingency table was constructed, based on unique FAERS reports (PRIMARYID), where a represented reports containing both meropenem and the target PT, b represented reports containing meropenem with other PTs, c represented reports containing the target PT with other drugs, and d represented reports containing neither meropenem nor the target PT. Signal detection was based on established criteria applied to the contingency table cell counts and derived statistics. PTs representing non-adverse-event concepts, including indications, medication errors, drug interactions, resistance-related terms, therapeutic failure, and product quality issues, were excluded before final signal interpretation. For the frequentist methods, a signal was considered present when a ≥ 3 and the lower limit of the 95% confidence interval (CI) of the ROR exceeded 1, and when a ≥ 3, PRR ≥ 2 and χ^2^ ≥ 4. For Bayesian methods, signals were considered positive when a ≥ 3 and EBGM05>2, and when a ≥ 3 and the lower limit of the information component 95% credibility interval (IC025) exceeded 0. PTs fulfilling all four predefined signal detection criteria (ROR, PRR, EBGM, and IC) were classified as positive signals. To reduce the risk of false-positive findings from multiple comparisons across PTs, a Bonferroni correction was applied to the *P*-values from PRR chi-square statistics for all 1,734 initially identified PTs, providing a conservative adjustment for PT-level comparisons. PTs with Bonferroni-corrected *P* values < 0.05 were considered statistically significant. Bayesian shrinkage-based measures, including EBGM and IC, were interpreted using their predefined lower-bound thresholds and were not subjected to additional Bonferroni correction. For age-stratified disproportionality analyses, separate 2 × 2 contingency tables were constructed within each age stratum using all FAERS reports from the corresponding age group as the reference population. All statistical formulas and thresholds are provided in Supplementary Table 1. The complete PT-level signal output, including the four-cell contingency table values (a, b, c, and d) and corresponding disproportionality metrics, is provided in Supplementary Table 3. For visualization of age-stratified signal distributions, PT-level and SOC-level counts across age groups were transformed into row-wise *Z* scores. Positive *Z*-score values indicate relatively enriched reporting within a given age group compared with the overall age distribution of the same PT or SOC, whereas negative values indicate relative underrepresentation. These *Z*-score transformations were used solely for visualization purposes and do not represent effect sizes or disproportionality estimates. For age-stratified analyses, a Bonferroni correction was applied separately within each age stratum, based on the number of PTs tested in that stratum.

### 2.5 Time-to-onset and Weibull parameter analysis

Time-to-onset (TTO) was defined as the interval between the reported date of adverse event occurrence and the initiation date of meropenem therapy. Only reports containing both valid treatment initiation dates and adverse event onset dates, with non-negative TTO values, were included. A total of 1,371 eligible reports were included in the overall TTO analysis. TTO was summarized using the median and interquartile range (IQR). Weibull distribution modeling was performed to characterize temporal reporting patterns within this subset. In addition to the overall TTO analysis, exploratory SOC-level TTO analyses were conducted using report–SOC pairs among reports with available date information. Each report could contribute to more than one SOC if adverse events from different SOC categories were recorded. SOCs with at least 20 report-SOC pairs were summarized using median TTO, IQR, and the proportions of reports occurring within 3, 7, and 10 days. Weibull parameters were estimated for SOCs with at least 30 report–SOC pairs. For Weibull fitting, TTO values of 0 day were treated as 0.5 days to allow continuous distribution modeling. Within the Weibull model, the scale parameter (α) reflects the temporal scale of reporting, whereas the shape parameter (β) characterizes the temporal reporting pattern. A β value less than 1 was interpreted as an early-reporting pattern, β approximately equal to 1 as a random-compatible or indeterminate reporting pattern, and β greater than 1 as a delayed-reporting pattern. Given the incomplete availability of date information in FAERS, both overall and SOC-level TTO analyses were interpreted as descriptive temporal reporting patterns rather than representative estimates for all meropenem-associated reports.

## 3 Results

### 3.1 Data selection workflow and construction of the analytical cohort

After drug name standardization and restriction to reports in which meropenem was designated as the primary suspect drug, 5,018 PT-level meropenem- related drug-event pairs were identified and used for the overall disproportionality analysis. Because a single FAERS report may contain more than one adverse event PT, these PT-level drug–event pairs corresponded to 4,431 unique meropenem-associated reports after merging the DEMO, DRUG, and REAC tables by PRIMARYID and deduplicating at the report level. All report-level analyses were subsequently conducted using analytical subsets derived from these 4,431 deduplicated reports. Specifically, demographic characterization, outcome analysis, and age-stratified analyses included 3,832 reports with complete age information; the HCP-only sensitivity analysis included 3,184 reports submitted by healthcare professionals according to the predefined FAERS reporter-type coding scheme; and time-to-onset (TTO) and Weibull parameter analyses included 1,371 reports with complete treatment initiation and adverse event onset date information.

### 3.2 Age-stratified demographic and reporting characteristics

Among the 3,832 reports with valid age information, the 60–79-year age group accounted for the largest number of reports (*n* = 1,311), followed by the 40–59-year group (*n* = 878), the < 18-year group (*n* = 677), the 18–39-year group (*n* = 502), and the ≥80-year group (*n* = 464; Table 1).

**Table 1:** Demographic and clinical characteristics of meropenem-related adverse event reports across age groups in the FAERS database.

Variable	<18 (*n* = 677)	18–39 (*n* = 502)	40–59 (*n* = 878)	60–79 (*n* = 1,311)	≥80 (*n* = 464)
Gender					
Female	293 (43.3%)	277 (55.2%)	383 (43.6%)	525 (40.0%)	206 (44.4%)
Male	372 (54.9%)	219 (43.6%)	480 (54.7%)	763 (58.2%)	246 (53.0%)
Missing	12 (1.8%)	6 (1.2%)	15 (1.7%)	23 (1.8%)	12 (2.6%)
Weight					
<50 kg	168 (24.8%)	32 (6.4%)	21 (2.4%)	51 (3.9%)	28 (6.0%)
>100 kg	2 (0.3%)	10 (2.0%)	46 (5.2%)	40 (3.1%)	10 (2.2%)
≥50 and < 100 kg	49 (7.2%)	86 (17.1%)	184 (21.0%)	323 (24.6%)	134 (28.9%)
Missing	458 (67.7%)	374 (74.5%)	627 (71.4%)	897 (68.4%)	292 (62.9%)
Reporter type					
Consumer	17 (2.5%)	19 (3.8%)	39 (4.4%)	62 (4.7%)	19 (4.1%)
Health professional	128 (18.9%)	133 (26.5%)	199 (22.7%)	242 (18.5%)	79 (17.0%)
Missing	219 (32.3%)	110 (21.9%)	160 (18.2%)	313 (23.9%)	92 (19.8%)
Pharmacist	59 (8.7%)	51 (10.2%)	153 (17.4%)	241 (18.4%)	125 (26.9%)
Physician	254 (37.5%)	189 (37.6%)	327 (37.2%)	453 (34.6%)	149 (32.1%)
Outcome					
Death	81 (12.0%)	63 (12.5%)	152 (17.3%)	237 (18.1%)	83 (17.9%)
Disability	5 (0.7%)	1 (0.2%)	5 (0.6%)	16 (1.2%)	1 (0.2%)
Hospitalization	177 (26.1%)	138 (27.5%)	277 (31.5%)	407 (31.0%)	157 (33.8%)
Life-threatening	61 (9.0%)	58 (11.6%)	111 (12.6%)	106 (8.1%)	39 (8.4%)
Other	353 (52.1%)	242 (48.2%)	333 (37.9%)	545 (41.6%)	184 (39.7%)
Non-serious/serious					
No	190 (28.1%)	95 (18.9%)	103 (11.7%)	175 (13.3%)	67 (14.4%)
Yes	487 (71.9%)	407 (81.1%)	775 (88.3%)	1,136 (86.7%)	397 (85.6%)
Non-fatal/fatal					
No	596 (88.0%)	439 (87.5%)	726 (82.7%)	1,074 (81.9%)	381 (82.1%)
Yes	81 (12.0%)	63 (12.5%)	152 (17.3%)	237 (18.1%)	83 (17.9%)
Year					
2004	6 (0.9%)	3 (0.6%)	6 (0.7%)	8 (0.6%)	2 (0.4%)
2005	6 (0.9%)	2 (0.4%)	2 (0.2%)	6 (0.5%)	1 (0.2%)
2006	5 (0.7%)	1 (0.2%)	8 (0.9%)	11 (0.8%)	3 (0.6%)
2007	13 (1.9%)	8 (1.6%)	8 (0.9%)	10 (0.8%)	8 (1.7%)
2008	14 (2.1%)	5 (1.0%)	11 (1.3%)	14 (1.1%)	11 (2.4%)
2009	26 (3.8%)	5 (1.0%)	6 (0.7%)	9 (0.7%)	1 (0.2%)
2010	13 (1.9%)	10 (2.0%)	8 (0.9%)	22 (1.7%)	5 (1.1%)
2011	60 (8.9%)	29 (5.8%)	22 (2.5%)	37 (2.8%)	11 (2.4%)
2012	50 (7.4%)	23 (4.6%)	22 (2.5%)	46 (3.5%)	20 (4.3%)
2013	20 (3.0%)	9 (1.8%)	21 (2.4%)	28 (2.1%)	5 (1.1%)
2014	22 (3.2%)	14 (2.8%)	17 (1.9%)	35 (2.7%)	14 (3.0%)
2015	29 (4.3%)	22 (4.4%)	35 (4.0%)	64 (4.9%)	12 (2.6%)
2016	46 (6.8%)	20 (4.0%)	22 (2.5%)	51 (3.9%)	11 (2.4%)
2017	34 (5.0%)	13 (2.6%)	56 (6.4%)	76 (5.8%)	25 (5.4%)
2018	26 (3.8%)	34 (6.8%)	64 (7.3%)	88 (6.7%)	39 (8.4%)
2019	34 (5.0%)	39 (7.8%)	55 (6.3%)	96 (7.3%)	38 (8.2%)
2020	41 (6.1%)	41 (8.2%)	77 (8.8%)	91 (6.9%)	31 (6.7%)
2021	39 (5.8%)	40 (8.0%)	80 (9.1%)	117 (8.9%)	33 (7.1%)
2022	27 (4.0%)	43 (8.6%)	70 (8.0%)	93 (7.1%)	29 (6.2%)
2023	43 (6.4%)	35 (7.0%)	69 (7.9%)	103 (7.9%)	43 (9.3%)
2024	58 (8.6%)	56 (11.2%)	103 (11.7%)	147 (11.2%)	55 (11.9%)
2025	65 (9.6%)	50 (10.0%)	116 (13.2%)	159 (12.1%)	67 (14.4%)

Regarding sex distribution, male reports predominated in most age groups except for the 18–39-year group, in which females accounted for a slightly higher proportion. Specifically, males represented 54.9% of reports in the < 18-year group, 54.7% in the 40–59-year group, 58.2% in the 60–79-year group, and 53.0% in the ≥80-year group, whereas females accounted for 55.2% of reports in the 18–39-year group.

With respect to reporter type, physician reports constituted a relatively high proportion across all age groups, ranging from 32.1 to 37.6%. Pharmacist reporting generally increased with age, reaching 26.9% in the ≥80-year group. Reports submitted by healthcare professionals were most common in the 18–39-year group (26.5%).

**Figure 2:**
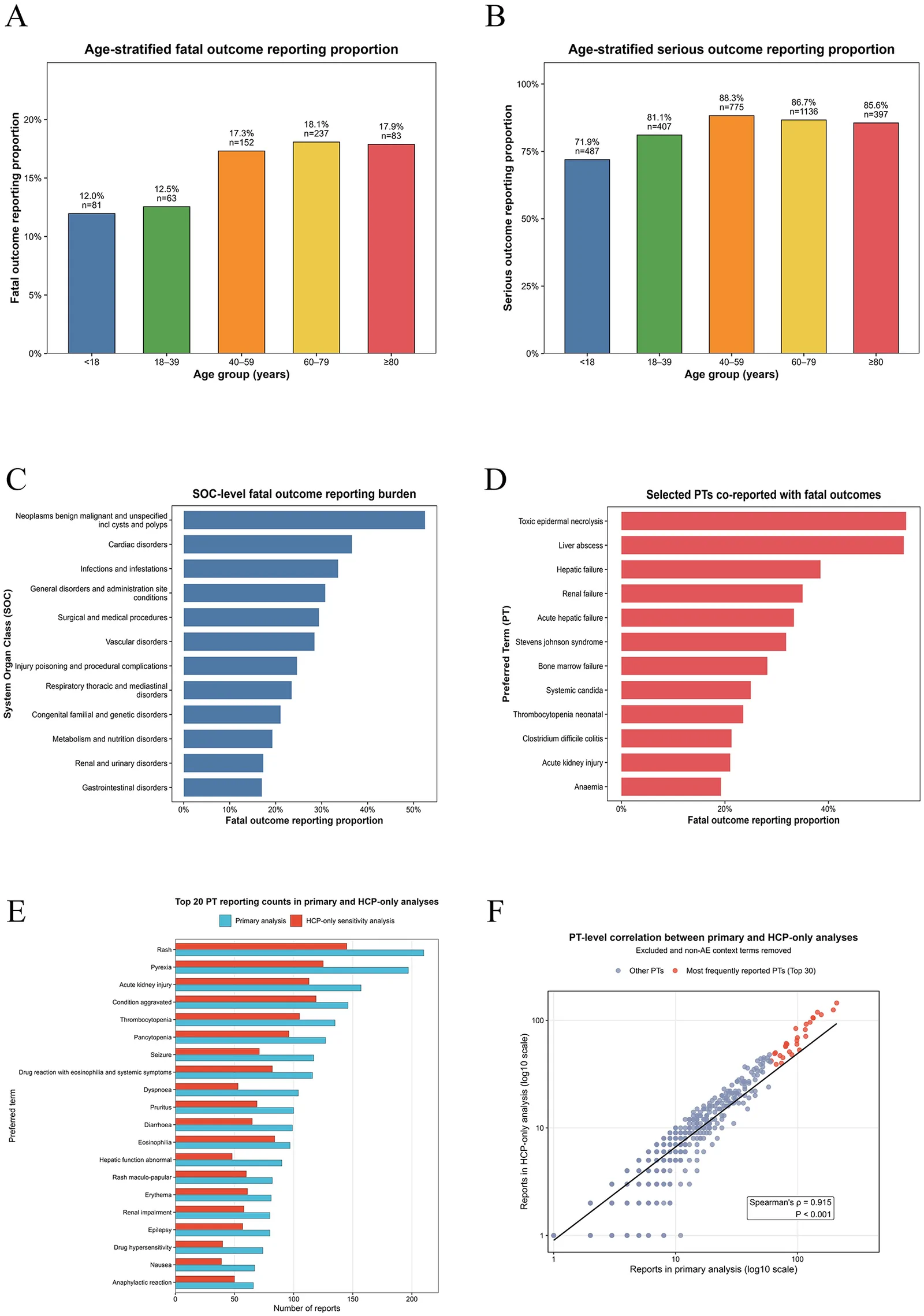
Outcome reporting patterns and HCP-only sensitivity analysis of meropenem-associated adverse event reports. **(A)** Age-stratified fatal outcome reporting proportions. **(B)** Age-stratified serious outcome reporting proportions. **(C)** SOC-level fatal outcome reporting burden. **(D)** Selected PTs co-reported with fatal outcomes. **(E)** Comparison of the 20 most frequently reported PTs between the primary analysis and the HCP-only sensitivity analysis. **(F)** PT-level reporting correlation between the primary analysis and the HCP-only sensitivity analysis. Each point represents a PT. Axes are displayed on a log10 scale. Red points indicate the most frequently reported PTs (Top 30), and blue points represent all other PTs. HCP, healthcare professional; PT, preferred term; SOC, system organ class.

Regarding outcomes, higher proportions of reports of serious outcomes were observed in older age groups. Serious outcomes accounted for 71.9% of reports in the < 18-year group, 81.1% in the 18–39-year group, 88.3% in the 40–59-year group, 86.7% in the 60–79-year group, and 85.6% in the ≥80-year group. The proportions of fatal outcomes were 12.0% and 12.5% in the < 18 and 18–39-year groups, respectively, and were 17.3%, 18.1%, and 17.9% in the 40–59, 60–79, and ≥80-year groups, respectively. Visualized age-related patterns of serious and fatal outcomes are presented in Figures 2A, B.

### 3.3 Overall meropenem-associated adverse event signals

**Figure 3:**
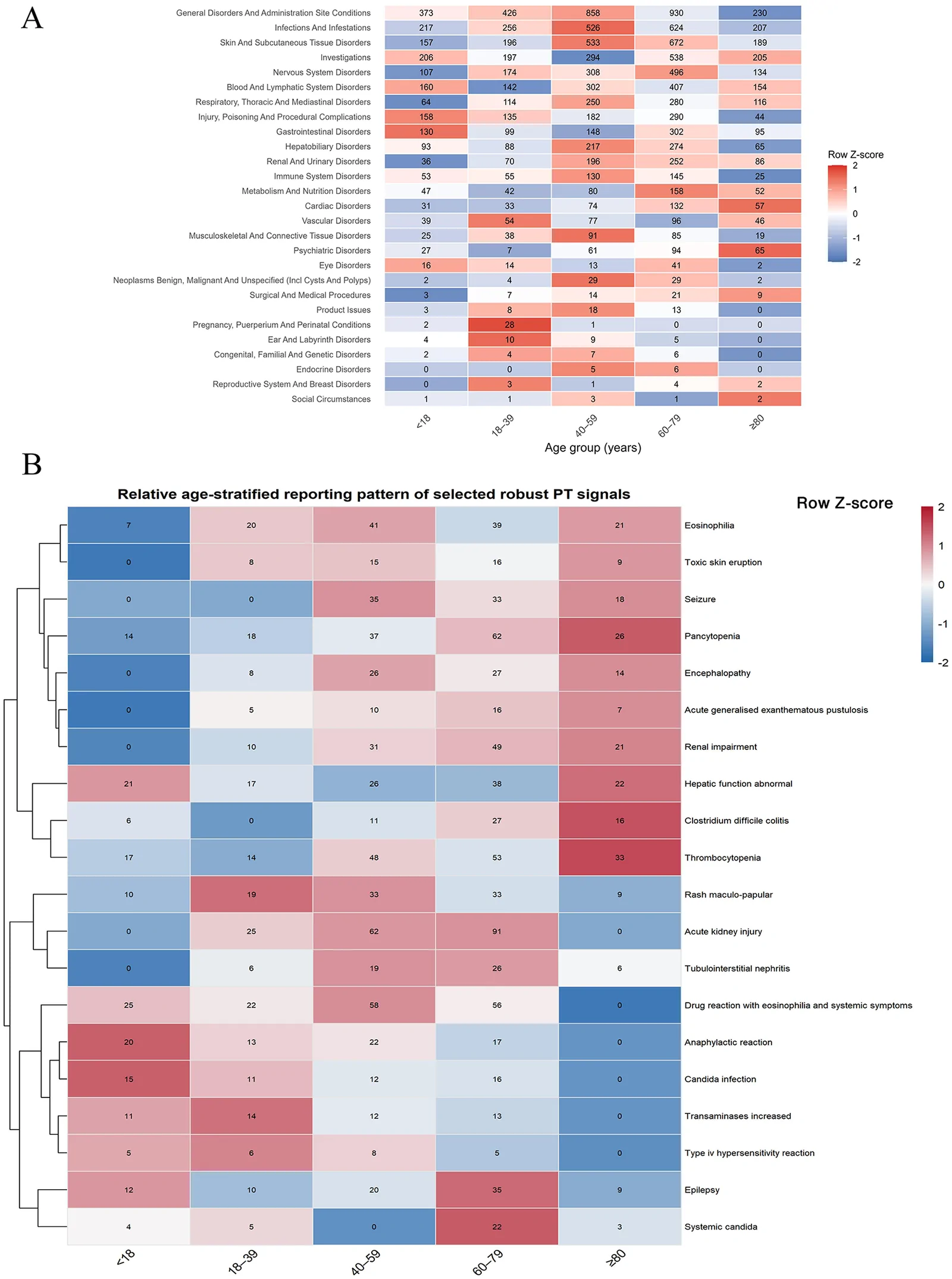
Age-stratified distribution of meropenem-related adverse event signals. **(A)** SOC-level heat map showing the age-graded distribution of SOC-level counties derived from robust pt-level signals. Colors indicate row-wise *Z* scores calculated from SOC-specific signal counts. **(B)** PT-level heatmap showing representative robust PT signals across age groups. Colors indicate row-wise *Z* scores calculated from PT-specific signal counts, with positive values indicating relatively enriched reporting within a given age group. PT, preferred term; SOC, system organ class.

A total of 1,734 unique PTs were initially identified. After exclusion of 875 PTs with fewer than three reports (a < 3) and 30 PTs (Supplementary Table 5) representing non-adverse-event concepts, 829 PTs remained for signal evaluation. Among these, 362 PTs met all four predefined signal-detection criteria (ROR, PRR, EBGM, and IC). Following Bonferroni correction, 254 PTs remained statistically significant and were retained as the final meropenem-associated adverse event signals (Table 2). At the SOC level, signals were primarily concentrated in infections and infestations, investigations, skin and subcutaneous tissue disorders, nervous system disorders, blood and lymphatic system disorders, and respiratory disorders (Figure 3A).

**Table 2:** Overall positive adverse event signals associated with meropenem identified using four pharmacovigilance algorithms and Bonferroni correction.

SOC	PT	Cases	ROR (95%CI)	PRR (χ^2^)	EBGM (EBGM05)	IC (IC025)	*P*-value	Bonferroni-adjusted *P*-value
Vascular disorders	Shock	49	7.75 (5.86–10.26)	7.73 (286.93)	7.72 (5.83)	2.95 (2.36)	6.7E-63	1.2E-59
	Cyanosis	25	5.69 (3.84–8.42)	5.68 (96.29)	5.67 (3.83)	2.5 (1.7)	1.2E-11	2.0E-08
	Haemodynamic instability^*^	13	6.36 (3.69–10.95)	6.35 (58.54)	6.34 (3.68)	2.67 (1.43)	2.7E-07	4.7E-04
Skin and subcutaneous tissue disorders	Drug reaction with eosinophilia and systemic symptoms	170	20.39 (17.53–23.73)	20.21 (3,092.34)	20.13 (17.3)	4.33 (3.96)	0.0E+00	0.0E+00
	Rash maculo-papular	104	16.7 (13.77–20.26)	16.61 (1,521.11)	16.56 (13.65)	4.05 (3.57)	0.0E+00	0.0E+00
	Toxic epidermal necrolysis	83	19.73 (15.89–24.48)	19.64 (1,462.35)	19.56 (15.76)	4.29 (3.69)	3.5E-75	6.0E-72
	Drug eruption	77	15.79 (12.61–19.75)	15.72 (1,058.15)	15.67 (12.52)	3.97 (3.39)	7.2E-63	1.2E-59
	Blister	62	3.82 (2.97–4.9)	3.81 (128.39)	3.81 (2.97)	1.93 (1.5)	3.8E-29	6.5E-26
	Rash erythematous	61	4.75 (3.7–6.11)	4.74 (180.07)	4.74 (3.68)	2.24 (1.79)	3.0E-40	5.3E-37
	Stevens–Johnson syndrome	57	9.31 (7.18–12.08)	9.28 (420.6)	9.27 (7.14)	3.21 (2.64)	1.1E-91	1.9E-88
	Toxic skin eruption	53	18.55 (14.16–24.31)	18.5 (874.11)	18.43 (14.07)	4.2 (3.41)	2.4E-47	4.2E-44
	Acute generalized exanthematous pustulosis	49	22.2 (16.76–29.4)	22.14 (984.39)	22.04 (16.64)	4.46 (3.55)	1.6E-47	2.8E-44
	Dermatitis bullous	32	15.45 (10.92–21.87)	15.43 (430.36)	15.38 (10.87)	3.94 (2.92)	7.2E-27	1.2E-23
	Purpura^*^	22	8.77 (5.77–13.33)	8.76 (151.03)	8.75 (5.76)	3.13 (2.11)	5.1E-14	8.8E-11
	Dermatitis exfoliative	19	13.38 (8.52–20.99)	13.36 (216.69)	13.33 (8.49)	3.74 (2.4)	1.7E-15	3.0E-12
	Rash morbilliform	18	22.96 (14.44–36.49)	22.94 (375.77)	22.83 (14.36)	4.51 (2.74)	9.8E-19	1.7E-15
	Petechiae^*^	16	5.31 (3.25–8.66)	5.3 (55.79)	5.3 (3.24)	2.41 (1.38)	1.3E-07	2.3E-04
	Dermatitis allergic	16	4.44 (2.72–7.25)	4.44 (42.54)	4.43 (2.71)	2.15 (1.18)	1.4E-06	2.3E-03
	Hypersensitivity vasculitis^*^	16	16.11 (9.86–26.32)	16.1 (225.74)	16.04 (9.82)	4 (2.39)	1.7E-14	3.0E-11
	Linear Iga disease^*^	15	28.39 (17.09–47.18)	28.37 (393.63)	28.2 (16.97)	4.82 (2.66)	3.4E-17	5.9E-14
	Dermatitis exfoliative generalized	14	14.53 (8.59–24.55)	14.52 (175.64)	14.47 (8.56)	3.86 (2.18)	2.9E-12	5.0E-09
	Fixed eruption^*^	12	22.69 (12.87–40.02)	22.68 (247.43)	22.57 (12.8)	4.5 (2.28)	6.3E-13	1.1E-09
	Exfoliative rash	8	10.24 (5.12–20.49)	10.23 (66.51)	10.21 (5.1)	3.35 (1.37)	1.7E-06	3.0E-03
	Erythema nodosum^*^	8	8.11 (4.05–16.23)	8.11 (49.77)	8.1 (4.05)	3.02 (1.22)	9.4E-06	1.6E-02
	Dermatosis^*^	8	46.06 (22.95–92.44)	46.04 (348.98)	45.59 (22.72)	5.51 (1.97)	1.8E-11	3.2E-08
	Subcorneal pustular dermatosis^*^	6	179.98 (79.59–406.99)	179.92 (1,027.02)	173.13 (76.56)	7.44 (1.64)	2.1E-12	3.7E-09
	Prurigo^*^	5	19.79 (8.22–47.64)	19.78 (88.78)	19.7 (8.18)	4.3 (1.08)	7.0E-06	1.2E-02
	Oculomucocutaneous syndrome^*^	5	44.44 (18.41–107.23)	44.43 (210.19)	44.01 (18.24)	5.46 (1.24)	1.4E-07	2.4E-04
	Sjs-ten overlap	4	29.85 (11.16–79.79)	29.84 (110.77)	29.65 (11.09)	4.89 (0.84)	1.2E-05	2.1E-02
	Mucocutaneous rash	3	70.5 (22.54–220.52)	70.48 (202.37)	69.43 (22.19)	6.12 (0.48)	1.3E-05	2.2E-02
Respiratory, thoracic and mediastinal disorders	Respiratory failure	100	4.72 (3.87–5.74)	4.7 (290.92)	4.69 (3.85)	2.23 (1.89)	2.0E-64	3.5E-61
	Hypoxia	36	3.63 (2.62–5.04)	3.63 (68.46)	3.62 (2.61)	1.86 (1.28)	4.8E-16	8.4E-13
	Respiratory distress	33	4.11 (2.92–5.78)	4.1 (77.46)	4.1 (2.91)	2.04 (1.41)	6.4E-18	1.1E-14
	Eosinophilic pneumonia^*^	32	38.2 (26.97–54.11)	38.13 (1,147.53)	37.82 (26.7)	5.24 (3.65)	6.8E-39	1.2E-35
	Bronchospasm	29	6.92 (4.8–9.96)	6.91 (146.36)	6.9 (4.79)	2.79 (2)	2.3E-15	4.0E-12
	Acute respiratory failure	22	3.99 (2.63–6.06)	3.99 (49.17)	3.98 (2.62)	1.99 (1.22)	1.0E-11	1.8E-08
	Acute respiratory distress syndrome	19	3.85 (2.45–6.03)	3.84 (39.96)	3.84 (2.45)	1.94 (1.1)	1.2E-06	2.1E-03
	Pulmonary hemorrhage^*^	14	6.08 (3.6–10.28)	6.08 (59.34)	6.07 (3.59)	2.6 (1.44)	1.6E-07	2.8E-04
	Eosinophilic pneumonia acute^*^	14	61.88 (36.51–104.88)	61.83 (826.69)	61.02 (36)	5.93 (2.86)	9.5E-21	1.6E-17
	Apnoea	11	4.85 (2.68–8.75)	4.84 (33.51)	4.84 (2.68)	2.27 (1.04)	2.6E-05	4.6E-02
	Respiratory acidosis^*^	8	7.21 (3.6–14.42)	7.2 (42.68)	7.2 (3.6)	2.85 (1.13)	2.2E-05	3.7E-02
	Pulmonary eosinophilia^*^	4	30.04 (11.24–80.31)	30.04 (111.54)	29.85 (11.16)	4.9 (0.84)	1.2E-05	2.1E-02
	Cheyne-stokes respiration^*^	4	56.11 (20.93–150.42)	56.1 (213.83)	55.43 (20.68)	5.79 (0.92)	1.0E-06	1.8E-03
	Respiratory muscle weakness^*^	4	39.99 (14.94–107.02)	39.98 (150.71)	39.64 (14.81)	5.31 (0.89)	3.9E-06	6.8E-03
Renal and urinary disorders	Acute kidney injury	217	3.66 (3.2–4.18)	3.62 (413.44)	3.62 (3.17)	1.86 (1.64)	2.4E-91	4.2E-88
	Renal impairment	127	5.27 (4.42–6.27)	5.24 (435.5)	5.23 (4.39)	2.39 (2.08)	8.6E-96	1.5E-92
	Tubulointerstitial nephritis^*^	59	9.67 (7.49–12.49)	9.64 (456.02)	9.62 (7.45)	3.27 (2.7)	2.6E-99	4.5E-96
	Anuria	23	9.03 (6–13.6)	9.02 (163.68)	9 (5.98)	3.17 (2.17)	7.6E-15	1.3E-11
	Nephropathy toxic^*^	19	6.14 (3.91–9.62)	6.13 (81.48)	6.12 (3.9)	2.61 (1.64)	9.5E-10	1.6E-06
	Oliguria	11	6.24 (3.45–11.27)	6.23 (48.26)	6.23 (3.45)	2.64 (1.28)	2.6E-06	4.5E-03
	Fanconi syndrome acquired^*^	6	11.12 (4.99–24.78)	11.12 (55.11)	11.09 (4.98)	3.47 (1.09)	2.2E-05	3.8E-02
Psychiatric disorders	Delirium	33	3.36 (2.39–4.73)	3.35 (54.51)	3.35 (2.38)	1.75 (1.15)	5.1E-13	8.8E-10
Pregnancy, puerperium and perinatal conditions	Premature baby^*^	36	3.97 (2.86–5.5)	3.96 (79.68)	3.96 (2.85)	1.99 (1.4)	1.9E-18	3.4E-15
	Premature delivery^*^	19	3.73 (2.38–5.85)	3.72 (37.85)	3.72 (2.37)	1.9 (1.07)	3.0E-09	5.2E-06
	Low birth weight baby^*^	17	6.86 (4.26–11.03)	6.85 (84.81)	6.84 (4.25)	2.77 (1.69)	1.4E-09	2.4E-06
Nervous system disorders	Seizure	171	3.47 (2.99–4.04)	3.45 (298.23)	3.45 (2.97)	1.79 (1.54)	2.7E-66	4.7E-63
	Epilepsy	113	13.11 (10.89–15.77)	13.03 (1,252.14)	13 (10.8)	3.7 (3.28)	1.1E-271	2.0E-268
	Encephalopathy^*^	77	11.13 (8.89–13.92)	11.08 (704.85)	11.06 (8.84)	3.47 (2.96)	4.0E-153	6.9E-150
	Depressed level of consciousness^*^	64	5.78 (4.52–7.39)	5.77 (251.92)	5.76 (4.51)	2.53 (2.07)	1.1E-55	1.9E-52
	Neurotoxicity^*^	46	9.45 (7.07–12.62)	9.43 (345.83)	9.41 (7.04)	3.23 (2.57)	7.1E-29	1.2E-25
	Generalized tonic-clonic seizure	41	5.62 (4.14–7.64)	5.61 (155.33)	5.61 (4.13)	2.49 (1.89)	1.2E-34	2.1E-31
	Status epilepticus	32	9.81 (6.93–13.88)	9.79 (252.19)	9.78 (6.91)	3.29 (2.45)	4.6E-21	8.0E-18
	Myoclonus	24	7.03 (4.71–10.49)	7.02 (123.76)	7.01 (4.7)	2.81 (1.92)	4.0E-13	6.9E-10
	Toxic encephalopathy^*^	22	15.4 (10.13–23.41)	15.38 (294.87)	15.33 (10.09)	3.94 (2.64)	6.1E-19	1.1E-15
	Altered state of consciousness^*^	22	3.55 (2.33–5.39)	3.54 (40.13)	3.54 (2.33)	1.82 (1.07)	8.5E-10	1.5E-06
	Hepatic encephalopathy^*^	16	5.7 (3.49–9.31)	5.7 (61.89)	5.69 (3.48)	2.51 (1.46)	5.2E-08	9.0E-05
	Partial seizures	12	8.1 (4.6–14.27)	8.09 (74.49)	8.08 (4.59)	3.01 (1.59)	6.1E-08	1.1E-04
	Convulsive threshold lowered	11	97.6 (53.7–177.39)	97.55 (1,029.09)	95.52 (52.56)	6.58 (2.58)	9.6E-19	1.7E-15
	Tonic clonic movements	7	19.06 (9.07–40.04)	19.05 (119.21)	18.97 (9.03)	4.25 (1.52)	1.3E-07	2.3E-04
	Febrile convulsion	5	14.78 (6.14–35.57)	14.78 (64.03)	14.74 (6.12)	3.88 (0.98)	2.8E-05	4.9E-02
Neoplasms benign, malignant and unspecified (Incl cysts and polyps)	Benign neoplasm of bladder^*^	4	107.27 (39.8–289.14)	107.25 (411.35)	104.8 (38.88)	6.71 (0.96)	8.3E-08	1.4E-04
Musculoskeletal and connective tissue disorders	Myopathy^*^	23	9.32 (6.19–14.03)	9.31 (170.21)	9.29 (6.17)	3.22 (2.2)	4.0E-15	6.9E-12
	Myositis^*^	14	5.77 (3.42–9.75)	5.77 (55.11)	5.76 (3.41)	2.53 (1.38)	3.0E-07	5.2E-04
Metabolism and nutrition disorders	Hypokalemia	37	2.82 (2.04–3.89)	2.82 (43.34)	2.82 (2.04)	1.49 (0.96)	1.2E-10	2.0E-07
	Hyperkalemia^*^	33	3.25 (2.31–4.57)	3.24 (51.19)	3.24 (2.3)	1.7 (1.11)	2.6E-12	4.5E-09
	Hyperamylasemia^*^	16	102.95 (62.71–169.01)	102.86 (1,578.32)	100.61 (61.29)	6.65 (3.17)	5.8E-27	1.0E-23
	Hypertriglyceridemia^*^	15	9.16 (5.52–15.21)	9.16 (108.77)	9.14 (5.51)	3.19 (1.88)	2.7E-10	4.8E-07
	Hypernatremia^*^	13	9.26 (5.37–15.95)	9.25 (95.47)	9.23 (5.36)	3.21 (1.77)	3.7E-09	6.4E-06
	Metabolic alkalosis^*^	13	29.05 (16.84–50.13)	29.03 (349.64)	28.85 (16.72)	4.85 (2.5)	3.2E-15	5.6E-12
	Hyperlipasemia^*^	8	50.39 (25.1–101.16)	50.36 (382.85)	49.83 (24.82)	5.64 (1.99)	9.1E-12	1.6E-08
	Vitamin K deficiency^*^	8	69.62 (34.63–139.97)	69.59 (532.69)	68.56 (34.1)	6.1 (2.04)	7.3E-13	1.3E-09
	Vitamin C deficiency^*^	5	98.26 (40.51–238.33)	98.23 (471.07)	96.18 (39.65)	6.59 (1.32)	2.9E-09	5.0E-06
	Electrolyte depletion^*^	4	28.19 (10.55–75.33)	28.18 (104.22)	28.01 (10.48)	4.81 (0.83)	1.5E-05	2.7E-02
Investigations	Alanine aminotransferase increased	66	3.88 (3.05–4.94)	3.87 (140.58)	3.87 (3.04)	1.95 (1.54)	8.4E-32	1.5E-28
	Aspartate aminotransferase increased	59	4.06 (3.15–5.25)	4.05 (135.67)	4.05 (3.14)	2.02 (1.57)	1.1E-30	1.9E-27
	C-reactive protein increased^*^	57	5.59 (4.31–7.25)	5.57 (213.85)	5.57 (4.29)	2.48 (1.99)	1.9E-47	3.4E-44
	Transaminases increased	52	7.93 (6.04–10.41)	7.91 (313.4)	7.9 (6.01)	2.98 (2.41)	1.2E-68	2.2E-65
	Clostridium test positive^*^	52	83.23 (63.24–109.53)	82.99 (4,136.88)	81.52 (61.94)	6.35 (4.62)	2.8E-79	4.8E-76
	Gamma-glutamyltransferase increased	46	7.35 (5.5–9.82)	7.34 (251.49)	7.33 (5.49)	2.87 (2.27)	2.9E-55	5.0E-52
	Liver function test abnormal	42	5.06 (3.74–6.85)	5.05 (136.44)	5.05 (3.73)	2.34 (1.77)	1.2E-30	2.1E-27
	Blood alkaline phosphatase increased	39	5.57 (4.07–7.63)	5.56 (145.7)	5.55 (4.06)	2.47 (1.86)	1.5E-32	2.6E-29
	White blood cell count increased	36	3.2 (2.31–4.44)	3.19 (54.24)	3.19 (2.3)	1.67 (1.12)	5.4E-13	9.3E-10
	Anticonvulsant drug level decreased	35	90.92 (65.04–127.09)	90.75 (3,045.9)	88.99 (63.67)	6.48 (4.21)	3.0E-55	5.2E-52
	Blood bilirubin increased	31	4 (2.81–5.7)	4 (69.7)	4 (2.81)	2 (1.36)	3.1E-16	5.4E-13
	International normalized ratio increased^*^	27	3.23 (2.22–4.72)	3.23 (41.54)	3.23 (2.21)	1.69 (1.03)	3.6E-10	6.2E-07
	Blood lactate dehydrogenase increased	27	6.01 (4.12–8.77)	6.01 (112.55)	6 (4.11)	2.58 (1.8)	5.1E-13	8.9E-10
	Blood creatine phosphokinase increased^*^	25	3.05 (2.06–4.51)	3.04 (34.32)	3.04 (2.06)	1.61 (0.93)	1.3E-08	2.3E-05
	Body temperature increased^*^	24	4.1 (2.74–6.12)	4.09 (56.06)	4.09 (2.74)	2.03 (1.29)	3.3E-13	5.8E-10
	Eosinophil count increased	23	10.01 (6.65–15.07)	10 (185.88)	9.98 (6.63)	3.32 (2.27)	9.1E-16	1.6E-12
	Anticonvulsant drug level below therapeutic	22	113.96 (74.63–174.01)	113.82 (2,400.34)	111.07 (72.74)	6.8 (3.65)	1.9E-37	3.3E-34
	Prothrombin time prolonged	17	10.36 (6.43–16.67)	10.35 (143.22)	10.33 (6.41)	3.37 (2.08)	2.8E-12	4.9E-09
	Procalcitonin increased^*^	15	45.8 (27.54–76.18)	45.76 (650.26)	45.32 (27.25)	5.5 (2.86)	3.3E-20	5.7E-17
	Activated partial thromboplastin time prolonged	15	10.96 (6.6–18.19)	10.95 (135.3)	10.93 (6.58)	3.45 (2.03)	2.4E-11	4.2E-08
	Amylase increased^*^	13	8.72 (5.06–15.02)	8.71 (88.58)	8.7 (5.05)	3.12 (1.72)	7.4E-09	1.3E-05
	Inflammatory marker increased^*^	9	6.4 (3.33–12.3)	6.39 (40.89)	6.39 (3.32)	2.67 (1.14)	1.7E-05	3.0E-02
	Staphylococcus test positive^*^	8	11.45 (5.72–22.93)	11.45 (76.1)	11.42 (5.71)	3.51 (1.44)	7.7E-07	1.3E-03
	Prothrombin level decreased^*^	7	27.35 (13.01–57.51)	27.34 (176.58)	27.18 (12.93)	4.76 (1.65)	1.2E-08	2.0E-05
	Enterococcus test positive^*^	7	36.23 (17.22–76.23)	36.22 (237.82)	35.94 (17.08)	5.17 (1.72)	1.7E-09	3.0E-06
	Apgar score low^*^	6	16.95 (7.6–37.79)	16.94 (89.69)	16.89 (7.57)	4.08 (1.28)	2.0E-06	3.5E-03
	Blood fibrinogen decreased^*^	6	20.03 (8.98–44.66)	20.02 (107.95)	19.94 (8.94)	4.32 (1.33)	7.9E-07	1.4E-03
	Human herpes virus 6 serology positive^*^	4	74.13 (27.6–199.11)	74.11 (283.89)	72.94 (27.16)	6.19 (0.94)	3.5E-07	6.1E-04
	Aspergillus test positive^*^	4	44.81 (16.73–119.97)	44.8 (169.61)	44.37 (16.57)	5.47 (0.9)	2.5E-06	4.4E-03
	Blood beta-D-glucan increased^*^	4	49.42 (18.45–132.39)	49.41 (187.68)	48.89 (18.25)	5.61 (0.91)	1.7E-06	3.0E-03
	Anti factor V antibody^*^	3	506.53 (153.65–1,669.85)	506.45 (1,362.01)	455.9 (138.29)	8.83 (0.46)	4.3E-08	7.4E-05
	Minimum inhibitory concentration increased^*^	3	569.85 (171.58–1,892.6)	569.75 (1,514.01)	506.56 (152.52)	8.98 (0.45)	3.1E-08	5.3E-05
Injury, poisoning and procedural complications	Contraindicated product administered^*^	29	3.19 (2.21–4.59)	3.18 (43.41)	3.18 (2.21)	1.67 (1.04)	1.3E-10	2.3E-07
	Documented hypersensitivity to administered product^*^	7	20.28 (9.65–42.61)	20.27 (127.67)	20.19 (9.61)	4.34 (1.55)	8.7E-08	1.5E-04
	Contraindicated product prescribed^*^	6	10.71 (4.8–23.86)	10.7 (52.66)	10.68 (4.79)	3.42 (1.07)	2.7E-05	4.7E-02
	Radiation fibrosis—lung^*^	4	230.84 (84.53–630.38)	230.79 (871.06)	219.71 (80.46)	7.78 (0.97)	4.2E-09	7.3E-06
Infections and infestations	Septic shock^*^	175	14.22 (12.25–16.5)	14.09 (2,123.17)	14.05 (12.1)	3.81 (3.49)	0.0E+00	0.0E+00
	Sepsis	139	4.25 (3.6–5.02)	4.23 (342.63)	4.22 (3.57)	2.08 (1.8)	8.6E-76	1.5E-72
	*Clostridium difficile* infection	70	10.51 (8.31–13.3)	10.48 (598.97)	10.46 (8.27)	3.39 (2.86)	3.1E-130	5.4E-127
	*Clostridium difficile* colitis	66	21.35 (16.76–27.21)	21.28 (1,269.74)	21.18 (16.63)	4.4 (3.67)	2.5E-62	4.3E-59
	Candida infection	62	10.46 (8.15–13.42)	10.42 (527.21)	10.4 (8.1)	3.38 (2.81)	1.2E-114	2.2E-111
	Pseudomembranous colitis	45	70.93 (52.82–95.24)	70.75 (3,047.29)	69.69 (51.89)	6.12 (4.38)	8.6E-66	1.5E-62
	Systemic candida	42	62.38 (45.99–84.61)	62.24 (2,496.58)	61.41 (45.27)	5.94 (4.23)	3.1E-59	5.4E-56
	Fungal infection	40	4 (2.93–5.45)	3.99 (89.69)	3.99 (2.93)	2 (1.44)	1.2E-20	2.2E-17
	Staphylococcal infection^*^	31	3.23 (2.27–4.59)	3.22 (47.51)	3.22 (2.26)	1.69 (1.08)	1.7E-11	2.9E-08
	Klebsiella infection^*^	26	18.66 (12.69–27.44)	18.64 (432.28)	18.57 (12.63)	4.21 (2.93)	3.8E-24	6.7E-21
	Fungemia	25	57.22 (38.56–84.92)	57.15 (1,362.06)	56.45 (38.04)	5.82 (3.6)	5.1E-35	8.9E-32
	Bacteremia	25	7.51 (5.07–11.12)	7.5 (140.72)	7.49 (5.06)	2.91 (2.02)	3.1E-14	5.4E-11
	Bronchopulmonary aspergillosis^*^	25	11.4 (7.7–16.88)	11.38 (236.23)	11.36 (7.67)	3.51 (2.45)	2.8E-18	4.8E-15
	Pseudomonas infection^*^	23	9.85 (6.54–14.83)	9.84 (182.2)	9.82 (6.52)	3.3 (2.25)	1.3E-15	2.2E-12
	Enterococcal infection^*^	19	14.23 (9.07–22.33)	14.21 (232.68)	14.17 (9.03)	3.83 (2.45)	5.9E-16	1.0E-12
	Endocarditis^*^	17	11.52 (7.16–18.55)	11.51 (162.78)	11.49 (7.13)	3.52 (2.18)	5.3E-13	9.3E-10
	Liver abscess^*^	16	17.11 (10.47–27.96)	17.1 (241.57)	17.04 (10.42)	4.09 (2.43)	7.0E-15	1.2E-11
	Fungal sepsis^*^	15	64.49 (38.73–107.38)	64.44 (923.78)	63.56 (38.17)	5.99 (2.97)	2.2E-22	3.8E-19
	Stenotrophomonas infection^*^	15	40.27 (24.22–66.97)	40.24 (568.98)	39.9 (23.99)	5.32 (2.81)	2.1E-19	3.7E-16
	Acinetobacter infection^*^	14	46.24 (27.31–78.3)	46.21 (613)	45.75 (27.02)	5.52 (2.77)	5.1E-19	8.8E-16
	Pneumonia bacterial^*^	13	5.28 (3.07–9.11)	5.28 (45.07)	5.28 (3.06)	2.4 (1.24)	2.0E-06	3.5E-03
	Enterobacter infection^*^	13	28.24 (16.36–48.72)	28.22 (339.18)	28.05 (16.26)	4.81 (2.48)	4.6E-15	7.9E-12
	Neutropenic sepsis^*^	13	6.14 (3.56–10.57)	6.13 (55.76)	6.12 (3.55)	2.61 (1.39)	4.0E-07	6.9E-04
	Superinfection	12	29.13 (16.51–51.39)	29.11 (323.66)	28.93 (16.4)	4.85 (2.4)	3.6E-14	6.2E-11
	Candida sepsis^*^	12	47.88 (27.11–84.58)	47.85 (544.78)	47.37 (26.81)	5.57 (2.57)	1.1E-16	1.9E-13
	Infectious pleural effusion^*^	12	25.87 (14.66–45.63)	25.85 (285.04)	25.71 (14.57)	4.68 (2.34)	1.4E-13	2.4E-10
	Encephalitis^*^	11	5.58 (3.09–10.08)	5.58 (41.27)	5.57 (3.08)	2.48 (1.18)	7.3E-06	1.3E-02
	Trichosporon infection^*^	10	89.77 (48–167.89)	89.72 (860.43)	88.01 (47.06)	6.46 (2.42)	8.2E-17	1.4E-13
	Staphylococcal bacteremia^*^	10	8.51 (4.57–15.82)	8.5 (66.08)	8.49 (4.56)	3.09 (1.47)	4.8E-07	8.4E-04
	Escherichia urinary tract infection^*^	10	7.93 (4.26–14.74)	7.92 (60.39)	7.91 (4.25)	2.98 (1.41)	9.1E-07	1.6E-03
	Clostridial infection^*^	9	11.06 (5.75–21.28)	11.06 (82.13)	11.03 (5.74)	3.46 (1.55)	2.1E-07	3.6E-04
	Brain abscess^*^	8	11.12 (5.56–22.27)	11.12 (73.51)	11.1 (5.54)	3.47 (1.42)	9.5E-07	1.6E-03
	Meningitis candida^*^	8	384 (186.6–790.25)	383.83 (2,817.43)	354.1 (172.07)	8.47 (2.13)	1.2E-18	2.2E-15
	Meningitis bacterial^*^	8	18.98 (9.48–38.01)	18.97 (135.64)	18.9 (9.44)	4.24 (1.7)	1.7E-08	3.0E-05
	Lung abscess^*^	7	11.67 (5.56–24.51)	11.67 (68.09)	11.64 (5.54)	3.54 (1.3)	3.3E-06	5.8E-03
	Klebsiella bacteremia^*^	7	28.32 (13.47–59.55)	28.31 (183.29)	28.14 (13.38)	4.81 (1.66)	9.2E-09	1.6E-05
	Nosocomial infection^*^	7	10.49 (4.99–22.02)	10.48 (59.9)	10.46 (4.98)	3.39 (1.24)	6.6E-06	1.1E-02
	Pneumonia necrotising^*^	7	39.95 (18.98–84.08)	39.93 (263.4)	39.59 (18.81)	5.31 (1.74)	8.9E-10	1.6E-06
	Gastroenteritis staphylococcal^*^	7	154.19 (72.59–327.53)	154.14 (1,030.16)	149.13 (70.21)	7.22 (1.89)	8.7E-14	1.5E-10
	Urinary tract infection fungal^*^	7	42.05 (19.98–88.53)	42.04 (277.87)	41.66 (19.79)	5.38 (1.75)	6.3E-10	1.1E-06
	Sepsis neonatal^*^	6	26.56 (11.9–59.27)	26.55 (146.68)	26.4 (11.83)	4.72 (1.42)	1.6E-07	2.7E-04
	Enterococcal sepsis^*^	6	25.52 (11.44–56.94)	25.51 (140.52)	25.37 (11.37)	4.67 (1.41)	2.0E-07	3.4E-04
	Clostridial sepsis^*^	6	59.09 (26.4–132.22)	59.07 (338.12)	58.32 (26.06)	5.87 (1.57)	1.5E-09	2.5E-06
	Hematological infection^*^	6	13.52 (6.06–30.13)	13.51 (69.31)	13.47 (6.05)	3.75 (1.18)	7.4E-06	1.3E-02
	Septic embolus^*^	5	20.97 (8.71–50.49)	20.97 (94.63)	20.87 (8.67)	4.38 (1.09)	5.3E-06	9.3E-03
	Intracranial infection^*^	5	186.86 (76.39–457.04)	186.8 (887.67)	179.49 (73.38)	7.49 (1.34)	1.3E-10	2.2E-07
	Corynebacterium infection^*^	5	35.18 (14.59–84.82)	35.17 (164.72)	34.91 (14.48)	5.13 (1.21)	4.4E-07	7.6E-04
	Rhinocerebral mucormycosis^*^	5	65.13 (26.94–157.48)	65.11 (311.2)	64.21 (26.56)	6 (1.29)	2.2E-08	3.8E-05
	Pyometra^*^	4	189.96 (69.86–516.52)	189.92 (721.62)	182.36 (67.07)	7.51 (0.97)	8.9E-09	1.5E-05
	Typhoid fever^*^	4	44.05 (16.45–117.93)	44.04 (166.64)	43.63 (16.29)	5.45 (0.9)	2.7E-06	4.7E-03
	Superinfection fungal	4	214.54 (78.7–584.88)	214.49 (811.8)	204.9 (75.16)	7.68 (0.97)	5.6E-09	9.7E-06
	Rotavirus infection^*^	4	25.22 (9.44–67.39)	25.22 (92.52)	25.08 (9.39)	4.65 (0.81)	2.4E-05	4.1E-02
	Pneumonia moraxella^*^	4	172.04 (63.39–466.91)	172 (655.3)	165.78 (61.08)	7.37 (0.97)	1.3E-08	2.3E-05
	Abscess bacterial^*^	4	37.22 (13.91–99.57)	37.21 (139.8)	36.92 (13.8)	5.21 (0.88)	5.2E-06	9.0E-03
	Cns ventriculitis^*^	4	43.11 (16.1–115.41)	43.1 (162.96)	42.71 (15.95)	5.42 (0.89)	2.9E-06	5.1E-03
	Candida endophthalmitis^*^	4	116.15 (43.05–313.38)	116.13 (445.2)	113.27 (41.98)	6.82 (0.96)	6.1E-08	1.1E-04
	Osteomyelitis bacterial^*^	4	51.95 (19.39–139.22)	51.94 (197.6)	51.37 (19.17)	5.68 (0.91)	1.4E-06	2.5E-03
	Renal cyst infection^*^	4	79.98 (29.76–214.97)	79.97 (306.53)	78.6 (29.25)	6.3 (0.95)	2.6E-07	4.5E-04
	Herpes simplex oesophagitis^*^	4	75.67 (28.17–203.28)	75.65 (289.85)	74.43 (27.71)	6.22 (0.94)	3.2E-07	5.6E-04
	Urinary tract candidiasis^*^	4	44.92 (16.77–120.27)	44.91 (170.04)	44.48 (16.61)	5.48 (0.9)	2.5E-06	4.3E-03
	Penicillium infection^*^	3	59.2 (18.95–184.94)	59.19 (169.44)	58.45 (18.71)	5.87 (0.47)	2.1E-05	3.7E-02
	Cutaneous mucormycosis^*^	3	79.98 (25.54–250.47)	79.97 (229.9)	78.6 (25.1)	6.3 (0.49)	8.9E-06	1.5E-02
	Endocarditis candida^*^	3	140.99 (44.69–444.87)	140.97 (404.42)	136.77 (43.35)	7.1 (0.5)	1.7E-06	2.9E-03
Immune system disorders	Anaphylactic reaction	85	5.51 (4.45–6.82)	5.49 (311.7)	5.48 (4.43)	2.45 (2.07)	8.8E-69	1.5E-65
	Anaphylactic shock	59	8.27 (6.41–10.69)	8.25 (375.41)	8.24 (6.38)	3.04 (2.5)	4.6E-82	8.0E-79
	Type IV hypersensitivity reaction^*^	27	34.23 (23.43–49.99)	34.18 (863.1)	33.93 (23.23)	5.08 (3.41)	8.0E-32	1.4E-28
	Haemophagocytic lymphohistiocytosis^*^	15	5.38 (3.24–8.93)	5.38 (53.4)	5.37 (3.24)	2.43 (1.35)	2.7E-07	4.8E-04
	Cross sensitivity reaction	14	21.87 (12.93–36.98)	21.85 (277.27)	21.75 (12.86)	4.44 (2.44)	1.3E-14	2.2E-11
	Jarisch–Herxheimer reaction^*^	13	56.36 (32.61–97.42)	56.33 (697.84)	55.65 (32.2)	5.8 (2.73)	7.4E-19	1.3E-15
	Serum sickness-like reaction^*^	8	46.53 (23.18–93.39)	46.51 (352.66)	46.05 (22.94)	5.53 (1.97)	1.7E-11	3.0E-08
Hepatobiliary disorders	Hepatic function abnormal^*^	121	11.87 (9.92–14.2)	11.8 (1,193.31)	11.77 (9.84)	3.56 (3.17)	3.8E-259	6.5E-256
	Drug-induced liver injury^*^	69	7.87 (6.22–9.98)	7.85 (411.84)	7.84 (6.19)	2.97 (2.49)	4.4E-90	7.7E-87
	Cholestasis	65	12 (9.4–15.31)	11.96 (651.13)	11.93 (9.35)	3.58 (3)	3.0E-141	5.1E-138
	Hepatitis^*^	52	7.26 (5.53–9.54)	7.25 (279.68)	7.24 (5.51)	2.86 (2.3)	2.0E-61	3.4E-58
	Liver disorder^*^	46	3.67 (2.74–4.9)	3.66 (88.9)	3.66 (2.74)	1.87 (1.37)	1.6E-20	2.7E-17
	Liver injury^*^	38	6.06 (4.41–8.33)	6.05 (160)	6.04 (4.39)	2.6 (1.96)	1.4E-35	2.4E-32
	Hepatic cytolysis^*^	38	12.08 (8.78–16.61)	12.05 (384.19)	12.02 (8.74)	3.59 (2.77)	8.0E-28	1.4E-24
	Hyperbilirubinemia	34	11.4 (8.14–15.97)	11.38 (321.24)	11.36 (8.11)	3.51 (2.64)	2.7E-24	4.7E-21
	Hepatocellular injury^*^	29	5.46 (3.8–7.87)	5.46 (105.47)	5.45 (3.79)	2.45 (1.72)	8.9E-24	1.5E-20
	Hepatotoxicity^*^	26	4.11 (2.8–6.04)	4.11 (61.1)	4.11 (2.79)	2.04 (1.32)	2.6E-14	4.5E-11
	Hypertransaminasemia	20	11.72 (7.56–18.18)	11.71 (195.39)	11.68 (7.53)	3.55 (2.32)	3.7E-15	6.4E-12
	Vanishing bile duct syndrome^*^	19	63.74 (40.52–100.27)	63.68 (1,156.05)	62.81 (39.93)	5.97 (3.29)	7.4E-28	1.3E-24
	Hepatitis cholestatic	18	10.94 (6.89–17.38)	10.93 (162.07)	10.91 (6.87)	3.45 (2.18)	2.6E-13	4.6E-10
	Acute hepatic failure	17	4.34 (2.7–6.99)	4.34 (43.64)	4.34 (2.69)	2.12 (1.19)	8.6E-07	1.5E-03
	Hepatitis acute	15	8.08 (4.87–13.41)	8.08 (92.84)	8.06 (4.86)	3.01 (1.76)	1.5E-09	2.5E-06
	Jaundice cholestatic	13	12.88 (7.47–22.2)	12.87 (141.93)	12.84 (7.45)	3.68 (2.03)	7.3E-11	1.3E-07
	Mixed liver injury^*^	10	13.53 (7.27–25.18)	13.53 (115.65)	13.49 (7.25)	3.75 (1.79)	7.0E-09	1.2E-05
	Hepatitis toxic^*^	9	12.17 (6.33–23.42)	12.17 (92.02)	12.14 (6.31)	3.6 (1.61)	9.5E-08	1.6E-04
	Hepatitis fulminant^*^	9	10.97 (5.7–21.11)	10.97 (81.34)	10.94 (5.69)	3.45 (1.54)	2.2E-07	3.9E-04
	Cholestatic liver injury^*^	8	14.18 (7.08–28.38)	14.17 (97.64)	14.13 (7.06)	3.82 (1.56)	1.6E-07	2.7E-04
	Hepatic infarction^*^	5	44.96 (18.63–108.51)	44.95 (212.77)	44.52 (18.45)	5.48 (1.25)	1.3E-07	2.3E-04
General disorders and administration site conditions	Pyrexia	290	2.92 (2.6–3.28)	2.89 (360.35)	2.89 (2.57)	1.53 (1.35)	6.1E-80	1.1E-76
	Multiple organ dysfunction syndrome^*^	156	12.32 (10.52–14.42)	12.22 (1,603.61)	12.19 (10.41)	3.61 (3.28)	0.0E+00	0.0E+00
	Treatment failure	85	3.58 (2.9–4.44)	3.57 (157.56)	3.57 (2.89)	1.84 (1.48)	1.4E-35	2.4E-32
	Face edema	30	6.22 (4.35–8.9)	6.21 (131.02)	6.2 (4.34)	2.63 (1.89)	1.2E-14	2.0E-11
	Hyperpyrexia	15	13.24 (7.97–21.98)	13.23 (169.1)	13.19 (7.95)	3.72 (2.18)	1.8E-12	3.1E-09
	Catheter site pain^*^	12	15.87 (9–27.97)	15.86 (166.49)	15.81 (8.97)	3.98 (2.08)	3.7E-11	6.5E-08
	Systemic inflammatory response syndrome	11	9.39 (5.2–16.97)	9.39 (82.25)	9.37 (5.18)	3.23 (1.63)	5.0E-08	8.6E-05
	Extravasation^*^	10	8.14 (4.38–15.14)	8.14 (62.49)	8.12 (4.37)	3.02 (1.43)	7.2E-07	1.2E-03
	Catheter site swelling^*^	6	21.51 (9.64–47.97)	21.5 (116.73)	21.4 (9.6)	4.42 (1.36)	5.2E-07	9.1E-04
	Polyserositis^*^	4	35.07 (13.11–93.8)	35.06 (131.35)	34.8 (13.01)	5.12 (0.87)	6.6E-06	1.1E-02
	Catheter site warmth^*^	4	85.22 (31.69–229.16)	85.2 (326.73)	83.65 (31.11)	6.39 (0.95)	2.0E-07	3.5E-04
Gastrointestinal disorders	Dysbiosis^*^	33	124.02 (87.74–175.31)	123.8 (3,913.37)	120.55 (85.28)	6.91 (4.24)	1.5E-56	2.6E-53
	Megacolon^*^	10	22 (11.82–40.95)	21.99 (199.37)	21.89 (11.76)	4.45 (2.04)	7.1E-11	1.2E-07
	Pancreatitis necrotising^*^	9	13.39 (6.96–25.77)	13.39 (102.87)	13.35 (6.94)	3.74 (1.66)	4.3E-08	7.4E-05
	Infantile diarrhea	8	160 (79.04–323.87)	159.93 (1,220.67)	154.54 (76.35)	7.27 (2.12)	1.1E-15	1.9E-12
	Neutropenic colitis^*^	6	10.65 (4.78–23.74)	10.65 (52.34)	10.63 (4.77)	3.41 (1.07)	2.8E-05	4.8E-02
	Abdominal compartment syndrome^*^	6	33.4 (14.96–74.58)	33.39 (187.16)	33.16 (14.85)	5.05 (1.47)	4.1E-08	7.1E-05
Ear and labyrinth disorders	Ototoxicity^*^	6	10.99 (4.93–24.48)	10.98 (54.32)	10.96 (4.92)	3.45 (1.08)	2.3E-05	4.0E-02
Cardiac disorders	Cardiac arrest	68	2.88 (2.27–3.65)	2.87 (82.83)	2.87 (2.26)	1.52 (1.13)	2.3E-19	4.0E-16
	Eosinophilic myocarditis^*^	6	29.26 (13.11–65.3)	29.25 (162.66)	29.07 (13.02)	4.86 (1.44)	8.9E-08	1.5E-04
	Cardiovascular insufficiency^*^	6	12.22 (5.48–27.24)	12.22 (61.65)	12.19 (5.47)	3.61 (1.14)	1.3E-05	2.2E-02
Blood and lymphatic system disorders	Pancytopenia^*^	181	11.56 (9.98–13.38)	11.45 (1,723.99)	11.43 (9.87)	3.51 (3.22)	0.0E+00	0.0E+00
	Thrombocytopenia	176	5.58 (4.81–6.48)	5.54 (655.06)	5.53 (4.77)	2.47 (2.21)	1.7E-143	3.0E-140
	Eosinophilia	133	27.32 (23.02–32.42)	27.12 (3,327.63)	26.97 (22.73)	4.75 (4.25)	3.3E-137	5.7E-134
	Leukopenia	73	5.1 (4.05–6.41)	5.08 (239.1)	5.07 (4.03)	2.34 (1.93)	4.8E-53	8.3E-50
	Bone marrow failure^*^	66	9.74 (7.64–12.4)	9.7 (514.31)	9.68 (7.6)	3.28 (2.75)	5.6E-112	9.7E-109
	Agranulocytosis	59	11.48 (8.89–14.83)	11.44 (561.1)	11.42 (8.84)	3.51 (2.91)	8.7E-122	1.5E-118
	Disseminated intravascular coagulation^*^	50	12.5 (9.47–16.51)	12.47 (526.22)	12.44 (9.42)	3.64 (2.94)	9.2E-37	1.6E-33
	Myelosuppression^*^	31	4.26 (2.99–6.06)	4.25 (77.05)	4.25 (2.99)	2.09 (1.44)	8.5E-18	1.5E-14
	Coagulopathy^*^	27	5.58 (3.83–8.14)	5.57 (101.24)	5.57 (3.82)	2.48 (1.71)	2.8E-12	4.8E-09
	Lymphopenia^*^	19	4.67 (2.98–7.33)	4.67 (54.76)	4.67 (2.98)	2.22 (1.33)	6.7E-08	1.2E-04
	Leukocytosis	19	3.72 (2.37–5.84)	3.72 (37.73)	3.72 (2.37)	1.89 (1.06)	3.2E-09	5.5E-06
	Thrombocytosis	18	15.4 (9.7–24.47)	15.39 (241.39)	15.34 (9.66)	3.94 (2.46)	8.8E-16	1.5E-12
	Methaemoglobinemia^*^	18	28.11 (17.68–44.68)	28.08 (467.19)	27.91 (17.56)	4.8 (2.87)	3.0E-20	5.2E-17
	Thrombocytopenia neonatal	17	153.58 (94.7–249.08)	153.44 (2,490.73)	148.47 (91.55)	7.21 (3.32)	1.9E-31	3.3E-28
	Hypocoagulable state^*^	10	33.56 (18.01–62.52)	33.54 (313.39)	33.3 (17.87)	5.06 (2.21)	1.2E-12	2.1E-09
	Hypereosinophilic syndrome^*^	9	61.72 (31.97–119.16)	61.69 (530.16)	60.88 (31.53)	5.93 (2.2)	7.7E-14	1.3E-10
	Haemorrhagic disorder^*^	8	24.77 (12.36–49.62)	24.75 (181.38)	24.63 (12.29)	4.62 (1.8)	2.3E-09	3.9E-06
	Monocytopenia^*^	6	205.69 (90.76–466.16)	205.62 (1,169.04)	196.79 (86.83)	7.62 (1.65)	9.7E-13	1.7E-09
	Bicytopenia^*^	6	11.44 (5.13–25.5)	11.44 (57.01)	11.41 (5.12)	3.51 (1.11)	1.9E-05	3.2E-02
	Factor V inhibition^*^	5	75.48 (31.19–182.69)	75.46 (361.4)	74.25 (30.68)	6.21 (1.3)	1.1E-08	1.8E-05
	Neutropenia neonatal	4	69.08 (25.73–185.44)	69.06 (264.3)	68.04 (25.35)	6.09 (0.94)	4.6E-07	8.1E-04
	Cyclic neutropenia	4	268.18 (97.82–735.22)	268.12 (1,005.35)	253.28 (92.39)	7.98 (0.96)	2.4E-09	4.1E-06
	Splenic embolism^*^	3	93.04 (29.66–291.83)	93.02 (267.63)	91.18 (29.07)	6.51 (0.49)	5.7E-06	9.8E-03

^*^Indicates adverse event PTs that are not explicitly labeled in the FDA prescribing information.

Ranked by report frequency, the most commonly reported PT signals included pyrexia, acute kidney injury, pancytopenia, thrombocytopenia, septic shock, seizure, drug reaction with eosinophilia and systemic symptoms (DRESS), multiple organ dysfunction syndrome, sepsis, and eosinophilia (Table 2). The overall signal distribution indicated that frequently reported signals involved infection progression, renal injury, and hematologic abnormalities, severe cutaneous adverse reactions, and immune-mediated events (Supplementary Figure 2B).

Several PTs with relatively low report frequency but disproportionately strong signal strength also warranted attention. For example, DRESS, toxic epidermal necrolysis, and acute generalized exanthematous pustulosis exhibited particularly strong disproportionality signals. These findings suggest that, in addition to common infection- and renal-related events, clinical monitoring should also pay attention to severe cutaneous reactions and immune hypersensitivity reactions (Table 2).

### 3.4 Time-to-onset and Weibull parameter analysis

**Figure 4:**
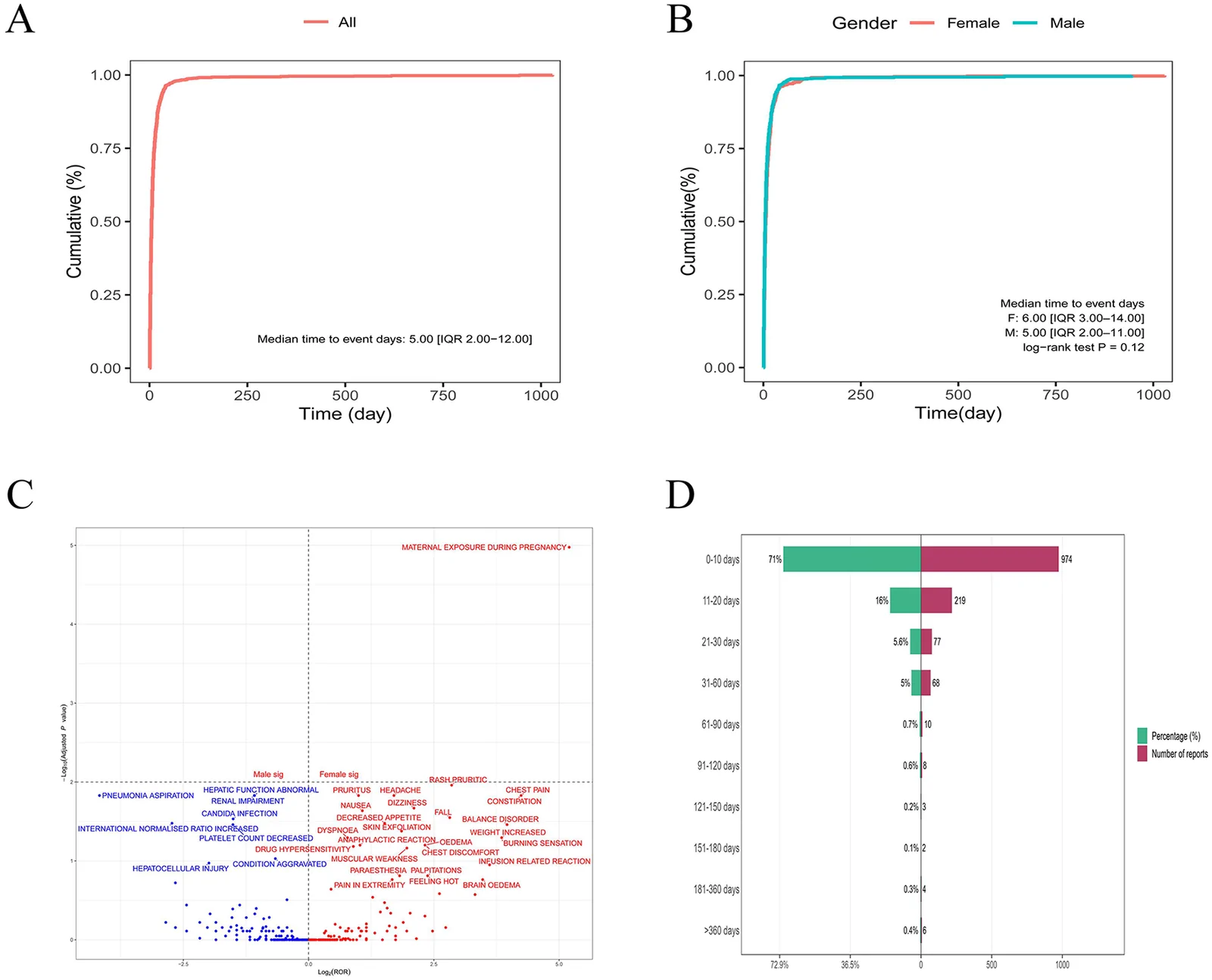
Overall characteristics of meropenem-related adverse events. **(A)** Cumulative distribution of time-to-onset among all meropenem-related adverse event reports. **(B)** Sex-stratified cumulative distribution of time-to-onset. **(C)** Volcano plot showing the distribution of adverse event signals according to signal strength and statistical significance. **(D)** Distribution of time-to-onset intervals for meropenem-related adverse events.

A total of 1,371 reports with complete treatment initiation and event onset dates were included in the TTO analysis. The median TTO was 5 days (IQR: 2–12 days), suggesting an early accumulation of reported events among date-complete reports (Table 3). The cumulative incidence curve demonstrated rapid accumulation of AEs shortly after treatment initiation, followed by a gradual plateau phase (Figure 4A). Following sex stratification, the median TTO was 6 days in females and 5 days in males, with broadly similar overall distributions (Figure 4B). Furthermore, PT signal distributions showed some variation between male and female reports (Supplementary Figure 1). Certain PTs exhibited relatively stronger disproportionality signals in either males or females; however, most PTs clustered within regions of limited difference, suggesting that the overall meropenem-associated AE spectrum was largely comparable between sexes, with only a minority of events potentially exhibiting sex-related differences (Figure 4C and Supplementary Figure 1). Weibull parameter analysis showed a scale parameter (α) of 10.49 (95% CI: 9.67–11.30) and a shape parameter (β) of 0.72 (95% CI: 0.70–0.75), indicating an early-reporting pattern among reports included in the TTO subset (Table 3). To further examine event-category-specific temporal patterns, exploratory SOC-level TTO analyses were performed using report–SOC pairs. The SOC-level results showed that several major SOCs had relatively early median TTO values, although temporal distributions and Weibull parameters varied across categories. These findings indicate that the overall Weibull estimate should be interpreted as a summary of the temporal reporting pattern rather than a uniform onset profile across all SOC categories. Detailed SOC-level TTO distributions and Weibull parameters are provided in Supplementary Table 4. Stratification by onset interval further supported the finding that meropenem-associated AEs were predominantly concentrated in the early accumulation of reported events during the initial treatment period (Figure 4D).

**Table 3:** Time-to-onset characteristics and Weibull parameter analysis of meropenem-related adverse events.

Cases	TTO	Weibull distribution		Failure type
	Median (IQR)	Scale parameter	Shape parameter	
		α (95% CI)	β (95% CI)	
1,371	5 (2–12)	10.49 (9.67–11.30)	0.72 (0.70–0.75)	Early-reporting pattern

TTO, time to onset; IQR, interquartile range; CI, confidence interval.

α represents the scale parameter of the Weibull distribution, and β represents the shape parameter. A shape parameter (β) < 1 indicates an early failure pattern, β = 1 indicates a random failure pattern, and β > 1 indicates a late failure pattern.

### 3.5 Age-stratified PT signal characteristics

Overall, age-related heterogeneity was observed at both the PT and SOC levels. A total of 458 PT signals were identified in the complete age-stratified analysis (Supplementary Table 2), with the highest number observed in the 60–79-year group, followed by the 40–59, 18–39, ≥80, and < 18-year groups (Supplementary Figure 2A).

In the < 18-year group, major signals included DRESS, bone marrow failure, hepatic function abnormal, anaphylactic reaction, and sepsis (Table 4). Relatively age-specific signals, including neutropenia neonatal, infantile diarrhea, impaired gastric emptying, and tooth discoloration, were also identified in this group (Supplementary Table 2), suggesting that hematologic, hepatobiliary, gastrointestinal, and pediatric-specific adverse events warrant particular attention among pediatric and adolescent patients.

**Table 4:** Age-stratified robust adverse event signals associated with meropenem in the FAERS database.

Age_group	PT	SOC	Case	ROR (95%CI)	PRR (χ^2^)	EBGM (EBGM05)	IC (IC025)	*P*-value	Bonferroni *P*-value
<18	Drug reaction with eosinophilia and systemic symptoms	Skin and subcutaneous tissue disorders	25	10.90 (7.34–16.20)	10.78 (220.52)	10.71 (7.21)	3.42 (2.39)	9.011E-18	4.48748E-15
<18	Bone marrow failure	Blood and lymphatic system disorders	24	16.96 (11.32–25.42)	16.77 (352.35)	16.60 (11.08)	4.05 (2.77)	2.22061E-21	1.10587E-18
<18	Hepatic function abnormal	Hepatobiliary disorders	21	16.54 (10.74–25.49)	16.38 (300.27)	16.22 (10.53)	4.02 (2.64)	1.0754E-18	5.3555E-16
<18	Anaphylactic reaction	Immune system disorders	20	6.24 (4.01–9.70)	6.18 (86.69)	6.16 (3.96)	2.62 (1.67)	2.8939E-10	1.44116E-07
<18	Sepsis	Infections and infestations	18	5.06 (3.18–8.06)	5.03 (57.96)	5.01 (3.15)	2.33 (1.38)	4.87921E-08	2.42985E-05
<18	Thrombocytopenia	Blood and lymphatic system disorders	17	4.34 (2.69–6.99)	4.31 (43.13)	4.30 (2.66)	2.10 (1.18)	9.2602E-07	0.000461158
<18	Multiple organ dysfunction syndrome	General disorders and administration site conditions	17	6.04 (3.75–9.75)	6.00 (70.67)	5.98 (3.71)	2.58 (1.54)	9.34153E-09	4.65208E-06
<18	Septic shock	Infections and infestations	16	8.50 (5.19–13.92)	8.44 (104.43)	8.40 (5.13)	3.07 (1.84)	2.2309E-10	1.11099E-07
<18	Candida infection	Infections and infestations	15	20.66 (12.39–34.44)	20.50 (274.85)	20.26 (12.15)	4.34 (2.47)	3.70858E-15	1.84687E-12
<18	Pancytopenia	Blood and lymphatic system disorders	14	6.31 (3.73–10.69)	6.27 (61.88)	6.25 (3.69)	2.64 (1.46)	1.08608E-07	5.4087E-05
<18	Hypertriglyceridemia	Metabolism and nutrition disorders	13	20.61 (11.90–35.68)	20.48 (237.83)	20.23 (11.68)	4.34 (2.31)	2.54117E-13	1.2655E-10
<18	Epilepsy	Nervous system disorders	12	4.54 (2.57–8.01)	4.51 (32.78)	4.50 (2.55)	2.17 (1.02)	2.25964E-05	0.01125299
<18	Transaminases increased	Investigations	11	7.71 (4.26–13.97)	7.67 (63.59)	7.64 (4.22)	2.93 (1.46)	3.56026E-07	0.000177301
<18	Rash maculo-papular	Skin and subcutaneous tissue disorders	10	7.46 (4.00–13.91)	7.43 (55.41)	7.40 (3.97)	2.89 (1.35)	1.59895E-06	0.000796275
<18	Thrombocytopenia neonatal	Blood and lymphatic system disorders	9	73.77 (37.76–144.14)	73.44 (614.59)	70.23 (35.94)	6.13 (2.21)	1.88882E-14	9.40631E-12
18–39	Pyrexia	General disorders and administration site conditions	56	3.87 (2.97–5.05)	3.80 (116.26)	3.80 (2.91)	1.93 (1.47)	1.71042E-26	8.51788E-24
18–39	Acute kidney injury	Renal and urinary disorders	25	5.80 (3.91–8.60)	5.74 (97.94)	5.73 (3.87)	2.52 (1.71)	8.61639E-12	4.29096E-09
18–39	Drug reaction with eosinophilia and systemic symptoms	Skin and subcutaneous tissue disorders	22	12.67 (8.32–19.30)	12.56 (233.46)	12.52 (8.22)	3.65 (2.45)	3.58881E-17	1.78723E-14
18–39	Neutropenia	Blood and lymphatic system disorders	21	4.55 (2.96–6.99)	4.52 (57.55)	4.51 (2.94)	2.17 (1.34)	2.33658E-08	1.16362E-05
18–39	Septic shock	Infections and infestations	21	17.41 (11.32–26.78)	17.25 (320.25)	17.18 (11.17)	4.10 (2.69)	3.65995E-19	1.82266E-16
18–39	Eosinophilia	Blood and lymphatic system disorders	20	27.20 (17.48–42.30)	26.96 (496.69)	26.78 (17.22)	4.74 (2.95)	5.10679E-22	2.54318E-19
18–39	Rash maculo-papular	Skin and subcutaneous tissue disorders	19	19.71 (12.53–30.99)	19.55 (332.89)	19.46 (12.37)	4.28 (2.69)	1.85626E-18	9.24416E-16
18–39	Pancytopenia	Blood and lymphatic system disorders	18	12.55 (7.89–19.98)	12.46 (189.24)	12.42 (7.81)	3.63 (2.29)	2.89678E-14	1.4426E-11
18–39	Hepatic function abnormal	Hepatobiliary disorders	17	13.30 (8.25–21.45)	13.21 (191.27)	13.17 (8.16)	3.72 (2.29)	5.95268E-14	2.96444E-11
18–39	Multiple organ dysfunction syndrome	General disorders and administration site conditions	16	9.87 (6.03–16.15)	9.80 (126.26)	9.78 (5.98)	3.29 (1.99)	2.54999E-11	1.2699E-08
18–39	Sepsis	Infections and infestations	15	5.54 (3.34–9.22)	5.51 (55.42)	5.51 (3.31)	2.46 (1.38)	1.95363E-07	9.72905E-05
18–39	Thrombocytopenia	Blood and lymphatic system disorders	14	4.98 (2.94–8.42)	4.95 (44.15)	4.95 (2.92)	2.31 (1.22)	1.70602E-06	0.000849598
18–39	Transaminases increased	Investigations	14	13.00 (7.68–22.01)	12.92 (153.60)	12.89 (7.61)	3.69 (2.10)	1.26288E-11	6.28914E-09
18–39	Generalized tonic-clonic seizure	Nervous system disorders	14	6.53 (3.86–11.06)	6.50 (65.09)	6.49 (3.84)	2.70 (1.50)	7.04616E-08	3.50899E-05
18–39	Anaphylactic reaction	Immune system disorders	13	4.87 (2.82–8.40)	4.84 (39.66)	4.84 (2.80)	2.27 (1.15)	4.9787E-06	0.002479395
40–59	Rash	Skin and subcutaneous tissue disorders	83	2.61 (2.10–3.24)	2.58 (80.80)	2.58 (2.07)	1.37 (1.02)	5.55417E-19	4.22117E-16
40–59	Acute kidney injury	Renal and urinary disorders	62	4.34 (3.38–5.58)	4.29 (156.91)	4.29 (3.34)	2.10 (1.66)	2.79267E-35	2.12243E-32
40–59	Drug reaction with eosinophilia and systemic symptoms	Skin and subcutaneous tissue disorders	58	24.10 (18.59–31.25)	23.80 (1,260.21)	23.67 (18.25)	4.56 (3.72)	8.82163E-58	6.70444E-55
40–59	Thrombocytopenia	Blood and lymphatic system disorders	48	6.38 (4.80–8.48)	6.32 (215.18)	6.32 (4.75)	2.66 (2.10)	1.45415E-47	1.10515E-44
40–59	Multiple organ dysfunction syndrome	General disorders and administration site conditions	43	13.52 (10.01–18.26)	13.40 (492.04)	13.36 (9.89)	3.74 (2.94)	3.8571E-33	2.9314E-30
40–59	Sepsis	Infections and infestations	43	5.63 (4.17–7.60)	5.58 (161.85)	5.58 (4.13)	2.48 (1.90)	4.41256E-36	3.35355E-33
40–59	Eosinophilia	Blood and lymphatic system disorders	41	31.51 (23.14-42.90)	31.23 (1,190.94)	31.00 (22.77)	4.95 (3.73)	5.71484E-46	4.34328E-43
40–59	Septic shock	Infections and infestations	38	11.85 (8.61–16.32)	11.76 (373.26)	11.73 (8.52)	3.55 (2.74)	1.72304E-27	1.30951E-24
40–59	Pancytopenia	Blood and lymphatic system disorders	37	9.58 (6.93–13.25)	9.51 (281.38)	9.49 (6.86)	3.25 (2.48)	1.02584E-23	7.7964E-21
40–59	Seizure	Nervous system disorders	35	3.32 (2.38–4.64)	3.31 (56.39)	3.30 (2.37)	1.72 (1.15)	1.90532E-13	1.44804E-10
40–59	Rash maculo-papular	Skin and subcutaneous tissue disorders	33	19.25 (13.65–27.13)	19.11 (563.95)	19.03 (13.50)	4.25 (3.14)	1.40534E-30	1.06806E-27
40–59	Renal impairment	Renal and urinary disorders	31	6.30 (4.42–8.97)	6.26 (137.08)	6.26 (4.39)	2.65 (1.91)	3.26077E-15	2.47818E-12
40–59	Cholestasis	Hepatobiliary disorders	27	17.86 (12.22–26.09)	17.76 (425.27)	17.68 (12.10)	4.14 (2.92)	1.71209E-24	1.30119E-21
40–59	Leukopenia	Blood and lymphatic system disorders	26	6.28 (4.27–9.23)	6.25 (114.50)	6.24 (4.24)	2.64 (1.83)	5.57115E-13	4.23408E-10
40–59	Hepatic function abnormal	Hepatobiliary disorders	26	9.29 (6.31–13.66)	9.24 (190.72)	9.22 (6.27)	3.20 (2.26)	7.72997E-17	5.87478E-14
60–79	Rash	Skin and subcutaneous tissue disorders	104	2.57 (2.12–3.12)	2.54 (97.96)	2.54 (2.09)	1.35 (1.04)	9.34432E-23	8.28841E-20
60–79	Acute kidney injury	Renal and urinary disorders	91	3.17 (2.58–3.91)	3.14 (133.36)	3.14 (2.55)	1.65 (1.31)	2.21906E-30	1.9683E-27
60–79	Septic shock	Infections and infestations	73	11.73 (9.30–14.78)	11.59 (704.92)	11.56 (9.17)	3.53 (3.00)	4.9717E-153	4.4099E-150
60–79	Multiple organ dysfunction syndrome	General disorders and administration site conditions	64	12.00 (9.37–15.35)	11.88 (635.89)	11.84 (9.25)	3.57 (2.98)	5.8571E-138	5.1953E-135
60–79	Pancytopenia	Blood and lymphatic system disorders	62	7.81 (6.08–10.03)	7.73 (363.22)	7.72 (6.01)	2.95 (2.44)	1.61209E-79	1.42992E-76
60–79	Drug reaction with eosinophilia and systemic symptoms	Skin and subcutaneous tissue disorders	56	18.79 (14.43–24.46)	18.62 (928.95)	18.52 (14.23)	4.21 (3.44)	4.05272E-50	3.59476E-47
60–79	Thrombocytopenia	Blood and lymphatic system disorders	53	3.81 (2.91–5.00)	3.79 (108.93)	3.79 (2.89)	1.92 (1.45)	6.81995E-25	6.0493E-22
60–79	Renal impairment	Renal and urinary disorders	49	4.71 (3.56–6.24)	4.68 (141.91)	4.68 (3.53)	2.23 (1.71)	6.42027E-32	5.69478E-29
60–79	Toxic epidermal necrolysis	Skin and subcutaneous tissue disorders	47	29.08 (21.80–38.80)	28.86 (1,253.69)	28.62 (21.46)	4.84 (3.76)	7.22132E-51	6.40531E-48
60–79	Sepsis	Infections and infestations	44	3.02 (2.24–4.06)	3.00 (58.91)	3.00 (2.23)	1.59 (1.09)	4.53043E-14	4.0185E-11
60–79	Blister	Skin and subcutaneous tissue disorders	41	7.55 (5.55–10.26)	7.50 (230.66)	7.49 (5.50)	2.90 (2.25)	1.0888E-50	9.65768E-48
60–79	Eosinophilia	Blood and lymphatic system disorders	39	17.37 (12.67–23.82)	17.26 (594.75)	17.18 (12.53)	4.10 (3.15)	3.37786E-34	2.99616E-31
60–79	Cardiac arrest	Cardiac disorders	38	4.11 (2.99–5.65)	4.09 (88.69)	4.08 (2.97)	2.03 (1.46)	2.17401E-20	1.92834E-17
60–79	Hepatic function abnormal	Hepatobiliary disorders	38	8.25 (5.99–11.35)	8.20 (239.80)	8.18 (5.94)	3.03 (2.32)	4.06013E-22	3.60133E-19
60–79	Respiratory failure	Respiratory, thoracic and mediastinal disorders	36	3.57 (2.57–4.96)	3.55 (66.14)	3.55 (2.56)	1.83 (1.26)	1.51891E-15	1.34727E-12
≥80	Thrombocytopenia	Blood and lymphatic system disorders	33	8.02 (5.68–11.32)	7.89 (198.39)	7.87 (5.57)	2.98 (2.21)	5.35592E-19	2.30304E-16
≥80	Pyrexia	General disorders and administration site conditions	32	4.07 (2.87–5.78)	4.02 (72.79)	4.01 (2.83)	2.01 (1.37)	6.60408E-17	2.83975E-14
≥80	Pancytopenia	Blood and lymphatic system disorders	26	10.12 (6.87–14.92)	9.99 (209.74)	9.95 (6.75)	3.31 (2.34)	1.16452E-17	5.00745E-15
≥80	*Clostridium difficile* infection	Infections and infestations	26	31.30 (21.20–46.21)	30.86 (742.09)	30.48 (20.65)	4.93 (3.30)	1.26887E-29	5.45612E-27
≥80	Hepatic function abnormal	Hepatobiliary disorders	22	14.13 (9.27–21.54)	13.97 (263.59)	13.89 (9.11)	3.80 (2.55)	4.10172E-18	1.76374E-15
≥80	Eosinophilia	Blood and lymphatic system disorders	21	22.92 (14.87–35.30)	22.66 (430.97)	22.46 (14.58)	4.49 (2.88)	1.61653E-21	6.95108E-19
≥80	Renal impairment	Renal and urinary disorders	21	4.17 (2.71–6.41)	4.13 (49.90)	4.13 (2.68)	2.04 (1.23)	7.82179E-12	3.36337E-09
≥80	Depressed level of consciousness	Nervous system disorders	20	8.81 (5.66–13.70)	8.72 (136.42)	8.69 (5.59)	3.12 (2.03)	7.1286E-13	3.0653E-10
≥80	Seizure	Nervous system disorders	18	6.30 (3.96–10.03)	6.25 (79.31)	6.24 (3.92)	2.64 (1.62)	1.83571E-09	7.89354E-07
≥80	Pseudomembranous colitis	Infections and infestations	17	,128.41 (78.67–209.60)	127.21 (2,022.16)	120.88 (74.06)	6.92 (3.28)	5.03646E-30	2.16568E-27
≥80	Septic shock	Infections and infestations	17	11.98 (7.42–19.33)	11.87 (168.58)	11.82 (7.32)	3.56 (2.20)	3.17473E-13	1.36514E-10
≥80	*Clostridium difficile* colitis	Infections and infestations	16	23.85 (14.55–39.12)	23.65 (343.87)	23.43 (14.29)	4.55 (2.63)	4.94244E-17	2.12525E-14
≥80	Drug eruption	Skin and subcutaneous tissue disorders	15	22.49 (13.50–37.47)	22.31 (302.65)	22.12 (13.27)	4.47 (2.52)	1.07178E-15	4.60865E-13
≥80	Clostridium test positive	Investigations	14	87.84 (51.43–150.02)	87.17 (1,150.98)	84.16 (49.28)	6.40 (2.92)	9.28511E-23	3.9926E-20
≥80	Encephalopathy	Nervous system disorders	14	14.73 (8.69–24.96)	14.62 (176.69)	14.54 (8.58)	3.86 (2.18)	2.54741E-12	1.09539E-09

In the 18–39-year group, pyrexia, acute kidney injury, DRESS, neutropenia, septic shock, and eosinophilia were commonly reported (Table 4), indicating that, in addition to infection-related events, immune-mediated reactions and hematologic abnormalities also deserve attention in young adults.

Within the 40–59-year group, rash, acute kidney injury, DRESS, thrombocytopenia, and multiple organ dysfunction syndrome were prominent signals (Table 4). This age group displayed a mixed profile characterized by cutaneous reactions, renal injury, hematologic abnormalities, and severe systemic events (Supplementary Table 2).

The 60–79-year age group had the highest number of reports. Common signals in this group included rash, acute kidney injury, septic shock, multiple organ dysfunction syndrome, and pancytopenia (Table 4). These signals were more frequently reported within this age stratum and primarily involved renal injury, infection-related events, and multiorgan dysfunction-related reports.

In the ≥80-year group, thrombocytopenia, pyrexia, pancytopenia, *Clostridium difficile* infection, hepatic function abnormal, and eosinophilia emerged as the principal signals (Table 4). Compared with other age groups, reports of *Clostridium difficile* infection, hematologic abnormalities, and hepatobiliary disorders appeared relatively more prominent among patients aged ≥80 years.

### 3.6 Age-enrichment patterns at the SOC level

The SOC-level heatmap demonstrated heterogeneous distribution of robust PT signals across age groups (Figure 3A). Infections and infestations, skin and subcutaneous tissue disorders, blood and lymphatic system disorders, hepatobiliary disorders, renal and urinary disorders, and nervous system disorders contributed substantially to the signal burden across multiple age strata. Colors represent row-wise *Z* scores calculated from SOC-specific signal counts across age strata, thereby reflecting relative age-enrichment patterns rather than absolute signal frequencies.

The PT-level heatmap further revealed age-related enrichment patterns among representative robust signals (Figure 3B). Colors represent row-wise *Z* scores derived from PT-specific counts across age groups, with positive values indicating relatively enriched reporting within a particular age stratum. Signals related to hypersensitivity reactions, hematologic abnormalities, renal injury, severe infections, and hepatobiliary disorders exhibited distinct age-dependent reporting patterns. For example, DRESS, anaphylactic reaction, and type IV hypersensitivity reaction were relatively enriched in younger age groups. In contrast, acute kidney injury, renal impairment, thrombocytopenia, pancytopenia, and *Clostridium difficile* infection were more prominent in middle-aged and older adults. These findings suggest that age-stratified analyses help identify clinically relevant differences in the spectrum of meropenem-associated adverse event reporting. For transparency, additional analyses of disease-context PTs that may be influenced by underlying infection severity, indication, or clinical status are presented in Supplementary Figures 2C, D. Although these PTs were not emphasized as core adverse event signals, age-related differences in their reporting patterns were also observed.

### 3.7 Signals associated with serious and fatal outcomes

Outcome analysis demonstrated a relatively high burden of serious outcomes among meropenem-associated reports. The proportions of serious outcomes exceeded 85% in the 40–59, 60–79, and ≥80-year groups (Figure 2B). Fatal outcome reporting proportions also increased with age and were highest in the 60–79-year group (18.1%), followed by the ≥80-year group (17.9%) and the 40–59-year group (17.3%; Figure 2A).

At the SOC-level, higher fatal outcome reporting proportions were observed for neoplasms benign malignant and unspecified (including cysts and polyps), cardiac disorders, infections and infestations, general disorders and administration site conditions and surgical and medical procedures (Figure 2C).

At the PT-level, toxic epidermal necrolysis, liver abscess, hepatic failure, renal failure, acute hepatic failure, Stevens–Johnson syndrome, bone marrow failure, systemic candidiasis, thrombocytopenia neonatal, *Clostridium difficile* colitis, acute kidney injury, and anemia were associated with relatively high fatal outcome reporting proportions (Figure 2D). PT-level analysis of serious outcome-associated signals identified drug-induced liver injury, *Clostridium difficile* infection, clostridium test positive, disease progression, fungal infection, toxic to various agents and confusional state as PTs associated with serious outcomes (Supplementary Figure 2F).

Analysis of death-associated signals further demonstrated strong disproportionality relationships between fatal outcomes and events such as multiple organ dysfunction syndrome, bronchopulmonary aspergillosis, death, cardiac arrest, and septic shock (Supplementary Figure 2E). Importantly, as FAERS is a spontaneous reporting database, these findings reflect report-level disproportionality signals rather than true incidence rates, relative risks, or causal associations.

### 3.8 HCP-only sensitivity analysis

The HCP-restricted sensitivity analysis included 3,184 reports from healthcare professionals. Compared with the primary analysis, the ranking of commonly reported PTs remained largely consistent in the HCP-only cohort. PTs such as rash, pyrexia, acute kidney injury, thrombocytopenia, pancytopenia, seizure, DRESS, dyspnoea, pruritus, diarrhea, eosinophilia, and hepatic function abnormal remained among the most frequently reported events in both the primary and HCP-only analyses (Figure 2E). PT-level report counts showed a strong positive correlation between the primary and HCP-only analyses, with a Spearman correlation coefficient of 0.915 (*P* < 0.001), indicating substantial consistency between the two datasets (Figure 2F).

## 4 Discussion

Unlike previous studies that primarily focused on the overall safety profile of carbapenem antibiotics, the present study further examined meropenem-associated adverse events with respect to age stratification, outcome severity, time to onset and reporting-source stability. The principal findings were as follows: meropenem-associated adverse event signals were more frequently reported among patients aged 40–79 years, and a higher proportion of serious and fatal outcome reports was observed among reports submitted for patients aged ≥40 years. Most adverse events occurred during the early treatment period, suggesting that the first 10 days after treatment initiation may represent a critical window for safety monitoring.

Age-stratified analyses showed that the burden of meropenem-associated signals did not increase linearly with age but was most concentrated among individuals aged 40–79 years. This finding suggests that clinical risk management should not focus exclusively on the very elderly (≥80 years) but should also pay close attention to hospitalized and critically ill patients aged >40 years, particularly those aged 40–79 years. Previous studies have indicated that the increased risk of adverse drug reactions among middle-aged and older adults is commonly associated with declining renal function, multimorbidity, polypharmacy, and reduced physiological reserve rather than age alone (21, 22). Lifespan pharmacokinetic studies have likewise suggested that meropenem dose optimization should primarily consider renal function rather than chronological age alone (23). Importantly, because age-stratified disproportionality analyses were conducted using age-specific FAERS reports as reference populations and FAERS lacks exposure denominators, these findings should be interpreted as relative reporting enrichment patterns rather than evidence of age-specific incidence or risk.

Distinct adverse event profiles were observed across age groups. In pediatric and adolescent patients, immune hypersensitivity reactions, hepatobiliary abnormalities, and hematologic disorders appeared relatively prominent. In contrast, middle-aged and older adults exhibited greater burdens of acute kidney injury, hematologic abnormalities, severe infection-related events, multiorgan dysfunction, and severe cutaneous reactions. These findings indicate that age-stratified analysis is not merely a demographic description but may help identify age-specific priorities for safety surveillance. From a clinical perspective, individualized risk assessment during meropenem therapy should therefore integrate patient age, renal function, underlying diseases, and treatment context.

The present study further demonstrated that serious and fatal outcomes were substantially more frequent among individuals aged ≥40 years than among younger patients. Because FAERS lacks information regarding true exposure denominators and disease severity, these findings should not be interpreted as evidence that meropenem directly increases the risk of severe outcomes. Nevertheless, from a pharmacovigilance perspective, the enrichment of serious and fatal outcomes among middle-aged and older patients remains clinically informative. Clinical monitoring should not focus solely on the most frequently reported events. Still, it should also prioritize high-risk events associated with serious or fatal outcomes, including multiple organ dysfunction syndrome, septic shock, respiratory failure, severe cutaneous adverse reactions, drug-induced liver injury, and neurologic events. Based on the present findings, clinicians may prioritize early monitoring within 10 days after meropenem initiation, particularly in patients aged ≥40 years, those with unstable renal function, and patients receiving intensive or combination antimicrobial therapy.

Renal- and nervous system-related signals were among the most clinically interpretable findings of this study. Because meropenem is primarily eliminated through renal excretion, changes in renal function may directly influence drug clearance and systemic exposure. The prescribing information recommends dose adjustment in patients with renal impairment and highlights the potential risk of seizures and other central nervous system adverse reactions. Recent studies on β-lactam-associated neurotoxicity have further demonstrated that renal dysfunction, elevated drug exposure, critical illness, and underlying neurological disease may all increase the risk of neurotoxic events (24, 25). In the present study, acute kidney injury, renal impairment, seizure, confusional state, depressed level of consciousness, and encephalopathy emerged as clinically relevant signals, suggesting that renal monitoring and neurological assessment should constitute essential components of meropenem safety management.

In addition, hematologic, hepatobiliary, and severe cutaneous adverse reactions deserve particular attention. Signals including thrombocytopenia, pancytopenia, neutropenia, eosinophilia, hepatic dysfunction, and drug-induced liver injury were identified in this study. Previous literature has suggested that carbapenems may be associated with platelet abnormalities (26). Although meropenem-related liver injury is considered relatively uncommon, case reports and ICU-based studies have indicated potential clinical significance (18, 27, 28). Furthermore, severe cutaneous reactions such as DRESS, toxic epidermal necrolysis (TEN), and toxic drug eruption, although not necessarily among the most frequent events, carry substantial clinical risk. The onset of β-lactam-associated DRESS may occur earlier than traditionally recognized (29), and antibiotics are recognized as important contributors to Stevens–Johnson syndrome and TEN (30, 31). Therefore, the occurrence of rash, fever, eosinophilia, hepatic dysfunction, or mucosal involvement during therapy should prompt timely evaluation for severe hypersensitivity reactions.

The TTO analysis of reports with available date information demonstrated a relatively short median onset time, and the Weibull analysis suggested an early-reporting pattern within this subset, indicating that reported events with available TTO data tended to occur during the early treatment period. The Weibull model has been widely applied in pharmacovigilance studies to describe temporal reporting patterns, with a shape parameter (β) < 1 generally interpreted as an early-failure or early-reporting pattern (32, 33). Although TTO data derived from spontaneous reporting systems may be affected by missing information, reporting delays, and right truncation, and should not be interpreted as a precise biological latency period (34), the observed clustering of reports within the first 10 days after meropenem initiation suggests that this period may warrant closer clinical attention. These findings may support heightened clinical awareness of neurological manifestations, renal dysfunction, hematological abnormalities, changes in liver function, gastrointestinal symptoms suggestive of *Clostridium difficile* infection, and cutaneous hypersensitivity reactions during the early treatment period. However, such observations represent reporting patterns and should not be interpreted as evidence of increased incidence or causal risk in specific patient populations.

Sex-stratified analysis did not reveal stable or systematic differences between males and females. Although a small number of PTs appeared relatively more prominent in one sex, the overall AE spectrum and TTO distributions were broadly comparable between sexes. Accordingly, in the present study, sex appeared to contribute less to the interpretation of meropenem-associated adverse events than age, organ function status, and temporal risk windows. Furthermore, an HCP-restricted sensitivity analysis demonstrated good agreement between the primary and healthcare professional-only analyses for high-frequency PTs and SOC distributions, with a strong PT-level correlation. These findings suggest that reports from non-healthcare professionals did not predominantly drive the principal signal structure identified in this study. Previous studies have similarly indicated that reporter type may influence reporting completeness and terminological accuracy; however, patient and consumer reports may also provide complementary safety information (35–37).

The present study employed four disproportionality analysis approaches-ROR, PRR, EBGM, and IC-combined with Bonferroni correction for signal screening. The use of multiple algorithms may reduce the instability associated with reliance on any single method and thereby enhance the robustness of signal interpretation. Nevertheless, disproportionality analyses remain susceptible to repeated reporting, indication-related confounding, database structure, comparator selection, and reporting bias and therefore require cautious interpretation (38–40). Consequently, the findings of this study should be interpreted as pharmacovigilance signals derived from FAERS reporting data rather than estimates of clinical incidence or causal risk.

This study has several limitations. First, as a spontaneous reporting database, FAERS is inherently subject to underreporting, duplicate reporting, selective reporting, and missing information and therefore cannot represent the true incidence of adverse events in real-world settings. Second, because FAERS lacks explicit drug exposure denominators, this study could not estimate the absolute or relative risk of meropenem-associated adverse events. Disproportionality analyses reflect report-level associations between drugs and adverse events rather than causal relationships, and may still be affected by reporting bias and database structure. In addition, meropenem is primarily prescribed for severe infections and critically ill patients, and infection severity, disease progression, organ dysfunction, and concomitant medications may all influence signal interpretation. FAERS also lacks key clinical information, including dosage, treatment duration, renal function, and detailed concomitant medication data, thereby limiting further adjustment analyses. Meropenem is primarily administered to patients with severe and life-threatening infections. Therefore, several identified signals, including sepsis, septic shock, multiple organ dysfunction syndrome, and acute kidney injury, may partly reflect the underlying disease process, infection severity, or critical illness rather than direct drug toxicity. Because FAERS lacks detailed clinical information, residual confounding by indication and disease severity cannot be excluded. Consequently, these findings should be interpreted as signals of disproportionality rather than evidence of causality. TTO analysis included only 1,371 reports with complete date information and may therefore be affected by missing dates, reporting delays, and selection bias. Likewise, age-stratified analyses were conducted only among reports containing valid age information, and their findings should therefore be interpreted as relative distribution patterns at the reporting level rather than true risk estimates. Despite these limitations, the present study leveraged large-scale real-world pharmacovigilance data to provide valuable evidence on the adverse event spectrum of meropenem and its age-related heterogeneity; these findings warrant further validation through clinical and prospective studies.

## 5 Conclusions

Meropenem-associated disproportionality signals involved multiple system organ classes and showed heterogeneous age-stratified reporting patterns and temporal characteristics. Signal reports were more frequently distributed among patients aged 40–79 years, while higher proportions of reported serious and fatal outcomes were observed among patients aged ≥40 years. TTO and Weibull analyses suggested an early-reporting pattern among reports with available date information. Collectively, these findings support age-stratified signal interpretation and early clinical monitoring as complementary components of meropenem pharmacovigilance, while emphasizing that FAERS-based signals represent reporting associations rather than causal evidence.

## Data Availability

Publicly available datasets were analyzed in this study. This data can be found here: the datasets presented in this study are available in online repositories: FAERS Dashboard (https://fis.fda.gov/extensions/FPD-QDE-FAERS/FPD-QDE-FAERS.html).

## Supplementary material

Supplementary Figure 1 Sex-stratified disproportionality analysis of selected meropenem-associated PT signals. Forest plot showing reporting odds ratios (RORs) and 95% confidence intervals (CIs) for female vs. male reports. The dashed line indicates ROR = 1. PT, preferred term; ROR, reporting odds ratio; CI, confidence interval.

Supplementary Figure 2 Additional age-stratified analyses of robust PT reporting signals. **(A)** Number of robust PT reporting signals across age groups. **(B)** SOC-level distribution of robust PT reporting signals across age groups. **(C)** Context-sensitive (Disease-context) PT reporting signals across age groups. Bubble size indicates the number of reports and color indicates EBGM05. **(D)** Heatmap of context-sensitive (Disease-context) PT reporting patterns. Numbers indicate report counts and colors represent row *Z*-scores. **(E)** Fatal outcome co-reporting analysis for selected PTs, including disease-context terms. Points indicate ROR estimates, horizontal lines indicate 95% CIs, and the dashed vertical line indicates ROR = 1. Red points indicate FDR < 0.05, and gray points indicate non-significant results. The *x*-axis is shown on a log scale. **(F)** Serious outcome co-reporting analysis for selected PTs, including disease-context terms. Points indicate ROR estimates, horizontal lines indicate 95% CIs, and the dashed vertical line indicates ROR = 1. Red points indicate FDR < 0.05, and gray points indicate non-significant results. The *x*-axis is shown on a log scale. PT, preferred term; SOC, system organ class; EBGM05, lower 95% confidence bound of the empirical Bayesian geometric mean.

Supplementary Table 1 Algorithms, formulas, and signal detection criteria used in pharmacovigilance analyses.

Supplementary Table 2 Age-stratified robust PT signals identified by four disproportionality algorithms and Bonferroni correction.

Supplementary Table 3 Complete list of meropenem-associated PT signals identified by four disproportionality algorithms and Bonferroni correction.

Supplementary Table 4 SOC-specific time-to-onset characteristics and Weibull distribution analysis of meropenem-associated adverse event reports. TTO, time-to-onset; SOC, system organ class; IQR, interquartile range. SOC-level analyses were conducted using report–SOC pairs among reports with available date information. Each report could contribute to more than one SOC if adverse events from different SOC categories were recorded. Weibull parameters were estimated for SOCs with at least 30 report–SOC pairs. For Weibull fitting, TTO values of 0 day were treated as 0.5 days to allow continuous distribution modeling. Weibull β < 1 was interpreted as an early-reporting pattern, β ≈ 1 as a random-compatible or indeterminate reporting pattern, and β > 1 as a delayed-reporting pattern.

Supplementary Table 5 Excluded preferred terms representing non-adverse-event concepts, including indications, medication errors, drug interactions, resistance-related terms, therapeutic failure, and product quality issues.
