# Supplementary material for: Real-world pharmacovigilance assessment of meropenem-associated adverse events: age stratification and time-to-onset analysis using FAERS
Source: Front Med (Lausanne). 2026 Aug 12;13:1890727. doi: 10.3389/fmed.2026.1890727 (PMC13506888; doi:10.3389/fmed.2026.1890727)
Supplement: Supplementary file 1 [file Data_Sheet_1.docx]

**Supplementary Table 1. Algorithms, formulas, and signal detection criteria used in pharmacovigilance analyses.**

| **Algorithm** | **Formula** | **Criteria** |
| --- | --- | --- |
| ROR | $ROR=\frac{a/c}{b/d}$ | a ≥ 3, |
|  | $95\%CI=e^{ln(ROR)\pm1.96\sqrt{\frac{1}{a}+\frac{1}{b}+\frac{1}{c}+\frac{1}{d}}}$ | Lower limit of 95% CI > 1 |
| PRR | $PRR=\frac{a}{a+b}/\frac{c}{c+d}$ | a≥3, PRR≥2, |
|  | $\chi^{2}=\frac{[(ad-bc)^{\wedge}2](a+b+c+d)}{[(a+b)(c+d)(a+c)(b+d)}$ | χ2≥4, |
|  | $95\%CI=eln(PRR)\pm1.96\sqrt{(\frac{1}{a}-\frac{1}{a+b}+\frac{1}{c}-\frac{1}{c+d})}$ |  |
| EBGM | $EBGM=a(a+b+c+d)/(a+c)(a+b)$ | a ≥ 3, |
|  | $EBGM05=e^{\ln(EBGM)-1.64(1/a+1/b+1/c+1/d)^{\wedge}0.5}$ | EBGM05>2 |
|  | $IC ={log}_{2}\frac{p(x,y)}{p(x)p(y)}={log}_{2}\frac{a(a+b+c+d)}{(a+b)(a+c)}$ | a ≥ 3, |
| IC | $E(IC)={log}_{2}\frac{(a+\gamma11)(a+b+c+d+a)(a+b+c+d+\beta)}{(a+b+c+d+\gamma)(a+b+\alpha1)(a+c+\beta1)}$ | IC025>0 |
|  | α1=β1=1; α=β=2; γ11=1 |  |

**Abbreviations:**

ROR, Reporting Odds Ratio, calculated from a 2×2 contingency table; PRR, Proportional Reporting Ratio, used to assess disproportionality between drug–event pairs; EBGM, Empirical Bayes Geometric Mean, an empirical Bayes–adjusted disproportionality measure derived from the MGPS algorithm; EBGM05, the lower one-sided 95% confidence bound of the EBGM (i.e., the 5th percentile of the posterior distribution); IC, Information Component, a Bayesian disproportionality metric based on the BCPNN method; IC025, the lower limit of the 95% credibility interval of the IC (i.e., the 2.5th percentile); CI, confidence interval;

χ², chi-square statistic, used to test the statistical significance of disproportionality in PRR analysis. a, number of reports of the target adverse event associated with the target drug; b, number of reports of other adverse events associated with the target drug; c, number of reports of the target adverse event associated with all other drugs; d, number of reports of other adverse events associated with all other drugs. α₁, β₁, α, β, γ₁₁, hyperparameters of the Bayesian prior distributions used in the BCPNN and MGPS models; default values were set as α1= β1 = 1, α = β = 2, and γ11 = 1.
